# What Is the Role of Motif D in the Nucleotide Incorporation Catalyzed by the RNA-dependent RNA Polymerase from Poliovirus?

**DOI:** 10.1371/journal.pcbi.1002851

**Published:** 2012-12-27

**Authors:** Hujun Shen, Hui Sun, Guohui Li

**Affiliations:** Laboratory of Molecular Modeling and Design, State Key Laboratory of Molecular Reaction Dynamics, Dalian Institute of Chemical Physics, Chinese Academy of Sciences, Dalian, China; University of Maryland, Baltimore, United States of America

## Abstract

Poliovirus (PV) is a well-characterized RNA virus, and the RNA-dependent RNA polymerase (RdRp) from PV (3D^pol^) has been widely employed as an important model for understanding the structure-function relationships of RNA and DNA polymerases. Many experimental studies of the kinetics of nucleotide incorporation by RNA and DNA polymerases suggest that each nucleotide incorporation cycle basically consists of six sequential steps: (1) an incoming nucleotide binds to the polymerase-primer/template complex; (2) the ternary complex (nucleotide-polymerase-primer/template) undergoes a conformational change; (3) phosphoryl transfer occurs (the chemistry step); (4) a post-chemistry conformational change occurs; (5) pyrophosphate is released; (6) RNA or DNA translocation. Recently, the importance of structural motif D in nucleotide incorporation has been recognized, but the functions of motif D are less well explored so far. In this work, we used two computational techniques, molecular dynamics (MD) simulation and quantum mechanics (QM) method, to explore the roles of motif D in nucleotide incorporation catalyzed by PV 3D^pol^. We discovered that the motif D, exhibiting high flexibility in either the presence or the absence of RNA primer/template, might facilitate the transportation of incoming nucleotide or outgoing pyrophosphate. We observed that the dynamic behavior of motif A, which should be essential to the polymerase function, was greatly affected by the motions of motif D. In the end, through QM calculations, we attempted to investigate the proton transfer in enzyme catalysis associated with a few amino acid residues of motifs F and D.

## Author Summary

The missing link between dynamics and structure or between dynamics and function of a protein has recently been paid much attention by many scientists since it has been recognized that a folded protein should be considered as an ensemble of conformations fluctuating in the neighborhood of its native state, instead of being pictured as a single static structure. Thus, to completely understand a protein and its functions, the dynamic features of the protein under a certain condition are required to be known. In this study, we performed atomistic MD simulations and QM calculations on the RNA-dependent RNA polymerase (RdRp) from poliovirus (PV), which is an important model system for gaining insight into the features of RNA and DNA polymerases. Through the computational studies of PV 3D^pol^, we aim at finding out valuable information about the dynamic properties of the enzyme and exploring the molecular mechanism of the phosphoryl transfer in nucleotide incorporation.

## Introduction

Poliovirus (PV) [1] is a well-characterized RNA virus and belongs to the family picornaviridae, in which we can find many famous viruses, such as rhinovirus, hepatitis A virus, foot and mouth disease virus, and so on [2]–[4]. The positive-sense RNA genome of PV, which is enclosed by a protein capsid shell, can be translated into a large polyprotein in a host cell, and then the large polyprotein is cleaved by viral proteases into a dozen of different proteins. Among those proteins, one can find the well-known RNA-dependent RNA polymerase (RdRp), termed 3D^pol^, which contains 461 amino acids residues [5]. The RNA-dependent RNA polymerase from poliovirus (PV 3D^pol^), serving as a target for developing antiviral drugs [6]–[9], has two-fold functions during RNA genome replication [10]. The first one is to catalyze the synthesis of the negative-sense complement of the positive-sense RNA genome, and the second one is to reproduce the positive-sense genome by using the negative-sense complement as template.

PV 3D^pol^ has been widely used as an important model system for understanding the structure-function relationships of RNA and DNA polymerases due to its simplicity and its retained activity in vitro without other proteins [6]–[9], [11], [12]. The crystal structure of PV 3D^pol^ has been solved [2], [5] and is similar to other RNA polymerases, containing three domains: fingers, palm and thumb, see Supporting Figure S1. The crystal structures of PV 3D^pol^ elongation complexes (EC) in different kinetic states, including the pre-chemistry and post-chemistry states, have been recently solved by Gong and Peersen [13]. These high quality crystal structures enable us to carry out theoretical studies on the dynamic properties of PV 3D^pol^. The complete structural description of PV 3D^pol^ has been given in the references [2], [5], [12], [13], and here we restate its structural elements for completeness.

**Figure 1:**
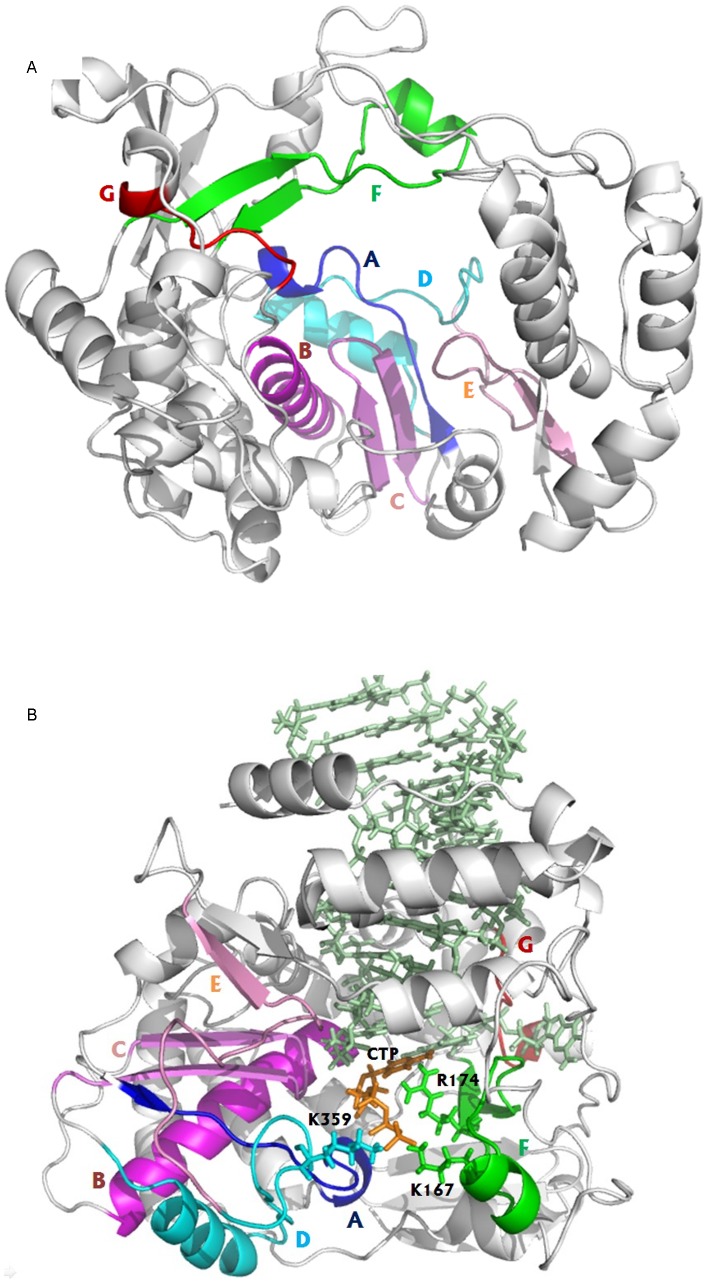
Cartoon representations of PV 3D^pol^ apo structure (A) and of the PV 3D^pol^-RNA-CTP complex structure (B). Motifs A, B, C, D, E, F and G are represented in blue, magenta, violet, cyan, pink, green and red, respectively. CTP, Lys359, Lys167 and Arg174 are indicated by orange, cyan, green and green sticks, respectively. The RNA primer and template are depicted by palegreen sticks.

The segment (residues 1–68) of PV 3D^pol^ is called the index finger which is mainly composed of random coils. Starting with the N-terminal glycine (Gly1) buried inside the base of the fingers, the index finger rises from the back of the palm subdomain, travels upwards to the top of the fingers and then makes a turn towards the thumb. In the previous studies of PV 3D^pol^ [14], the positively correlated motions were observed between amino acid residues of the fingers and those of the thumb. Following the turn, the index finger travels back toward the base of the fingers, and a hydrogen bonding interaction exists between Gly1 and Ser64. The segment (residues 69–95) of the palm, forming a long α-helix, is inserted between the index finger and the segment (residues 96–149) of the pinky finger. One segment (residues 96–149) of the pinky finger, having high α-helical contents and containing the structural motif G (residues 113–120), is followed by the ring finger (residues 150–179) to which the motif F (residues 153–178) belongs. The EC structures of PV 3D^pol^ [13] show that a coil segment of the pinky finger packs into the RNA major groove. Another segment (residues 180–190) of the pinky finger is succeeded by the segment (residues 191–268) of the palm where we can find the motif A (residues 229–240). The middle finger (residues 269–285) precedes to the last segment (residues 286–380) of the palm that contains several important structural motifs, such as motifs B (residues 293–312), C (residues 322–335), D (residues 338–362), and E (residues 363–380), see Figure 1. One should note that the five structural motifs A, B, C, D and E of the palm form the core subunit of the RNA polymerase. The last segment (residues 381–461) of PV 3D^pol^, beginning with the C-terminus of motif E, is the alpha-helix rich thumb subdomain that is responsible for the interaction with RNA primer/template. The EC structures of PV 3D^pol^ [13] indicate that an alpha helix of the thumb inserts into the RNA minor groove.

Recently, many experimental studies of PV 3D^pol^ revealed the role of viral RdRp fidelity during genome replication [6], [11], [15]–[19], showing that a small increase or decrease in viral polymerase fidelity could result in reduced viral population fitness. More specifically, viral pathogenesis is able to be attenuated by enhancing polymerase fidelity whereas a decrease in polymerase fidelity can force the virus into error catastrophe (lethal mutagenesis) [6], [20], [21]. The elementary steps of nucleotide incorporation catalyzed by RNA and DNA polymerase have been proposed as the following: (1) a nucleotide binds to polymerase; (2) a pre-chemistry conformational change; (3) the chemistry step of nucleotide incorporation; (4) a post-chemistry conformational change; (5) a pyrophosphate (PPi group) is released; (6) the translocation of nucleic acid. The studies of the Klenow fragment and HIV-1 RT DNA polymerases suggested that the pre-chemistry conformational change (step 2) should contribute to DNA polymerase fidelity [22]–[24], which was supported by the study of Tsai and Johnson [25]. However, other researchers believe that the chemistry step (step 3) should dictate nucleotide misincorporation rate [26]. Through the kinetic studies of nucleotide incorporation catalyzed by PV 3D^pol^, Arnold and Cameron proposed that the chemistry step should be rate limiting in the presence of Mn^2+^ [17] while the pre-chemistry and chemistry steps both contributed to polymerase fidelity in the presence of Mg^2+^ [16]. Thus, computational studies of dynamics in RNA and DNA polymerases may complete our understanding of polymerase fidelity. In fact, the dynamics of an enzyme has a substantial impact on its functional behavior [27]–[30]. Specifically, the structural motif D of PV 3D^pol^ has been recently considered as an important dynamic element that influences nucleotide incorporation [14], [31], but the roles of motif D are less well studied so far.

RNA and DNA polymerases catalyze nucleotide incorporation by the two-metal ion mechanism, as proposed by Steitz [32], [33]. In this mechanism, one metal (metal A), which is coordinated by two conserved acidic residues of motifs A and C, the primer terminus 3′-OH, as well as the triphosphate moiety of incoming nucleotide, facilitates enzyme catalysis through lowering pKa of the primer 3′-OH under physiological condition; another metal (metal B), which is coordinated by an acidic residue of motif A, the β- and γ-phosphate of incoming nucleotide, serves to orient the triphosphate of the incoming nucleotide as well as to facilitate the release of pyrophosphate. In addition, Cameron et. al [34] suggested that two protons involved in nucleotidyl transfer. According to this mechanism, one proton is donated from the primer terminus 3′-OH, but it is unclear where this proton is transferred to. It has been proposed that the protonation of the pyrophosphate leaving group is an important chemical step, but it is still unclear whether the residue Lys359 [35] of motif D or the residue Arg174 [13] of motif F serves as the proton donor. In addition, if the residue Lys359 serves as the proton donor, it is unclear whether it directly or indirectly donates the proton.

Molecule dynamics (MD) simulation has been widely used to reveal protein motions that are essential to protein functions [36]–[39]. Through molecular dynamics simulation carried out on PV 3D^pol^, we obtained valuable information about the dynamic properties of PV 3D^pol^ in solution and we observed that the PV 3D^pol^ was stabilized by the binding of RNA primer/template. However, the motif-D loop, containing Lys359, retained high flexibility even in the presence of RNA, suggesting that the dynamics of the loop may be important for enzyme catalysis. Through the MD study, we have showed the role of motif D in the pre-chemistry conformational change step (step 2) of nucleotide incorporation. In order to explore the role of motif D in the chemistry step (step 3), quantum mechanics (QM) calculations have been employed. Our results suggested that the hydrogen bonding interaction between the residue Arg174 of motif F and the incoming nucleotide could facilitate enzyme catalysis, and the residue Lys359 of motif D might indirectly donate a proton to the PPi leaving group during enzyme catalysis.

## Methods

### Molecular Dynamics (MD) Simulation

The starting apo structure of PV 3D^pol^ was obtained from the crystal structure (PDB code: 1RA6 [2]) deposited in the protein data bank. The initial structure of the 3D^pol^ elongation complex for MD simulations was obtained from the crystal structure (PDB code: 3OL7) [13], and then was edited in the program PYMOL [40] by removing components except for chain A (protein), chain B (RNA template), chain C (RNA primer), two Mg^2+^ ions and crystal waters. Thereafter, in the 3D^pol^-RNA complex, the RNA primer/template was further trimmed by removing unpaired nucleotides outside the polymerase as well as by deleting the cytosine nucleotide of RNA primer (see Supporting Figure S2a) since the crystal structure (PDB code: 3OL7) reflects a state of the post-chemistry step (step 4) of nucleotide incorporation. Then the nucleotide sequences retained in the truncated RNA primer and template are given in Supporting Figure S2b. Finally, the rCTP molecule was built into the 3D^pol^-RNA complex based on the crystal structure (PDB codes 3OLB) [13] containing 2′,3′-Dideoxy-CTP. One should note that the crystal structure (PDB code: 3OLB) reflects a state of the pre-chemistry step (step2) of nucleotide incorporation, and the heavy atom RMSD value between the two crystal structures (PDB codes: 3OLB and 3OL7), reflecting the pre-chemistry and post-chemistry states, was measured of 1.04 Å. In fact, the large global conformational change was not observed for the NTP binding to PV 3D^pol^ because the RMSD value between the two 3D^pol^-RNA complexes with NTP binding (PDB code: 3OLB) and without NTP binding (PDB code: 3OL6) was measured of less 1.5 Å. This implies that the MD simulation on the nano-second time scale may be suitable to study this system.

All molecular dynamics (MD) simulations were performed using the AMBER99SB force field in AMBER10 simulation package [41]. Each structure was immerged in a truncated octahedron solvent box filled with TIP3P water molecules [42] and then was neutralized with sodium ions. A minimal distance of 15 Å between the surface of each simulated structure and the edge of solvent box was employed. The energy minimization with constraining no-solvent atoms was followed by the minimization of the whole system for a few thousand steps. Each system was slowly heated to 300 K from 100 K over a period of 100 ps under NVT condition, and the Berendsen thermostat [43] was used to control the temperature. Subsequently, a 100 ps NVT dynamics was performed before the production run under NPT condition. The integration time step was set to 2 fs since the SHAKE algorithm [44] was employed to constrain all bonds associated with hydrogen atoms, and a cutoff value of 10 Å was set for the nonbonded interactions during the NPT simulation. The Particle Mesh Ewald method [45] was used for electrostatic interactions. For each system, five independent MD simulations have been carried out and each simulation was performed for at least 20 ns, so the total simulation time for each system reached to 100 ns.

### Quantum Mechanics (QM) Calculations

**Figure 2:**
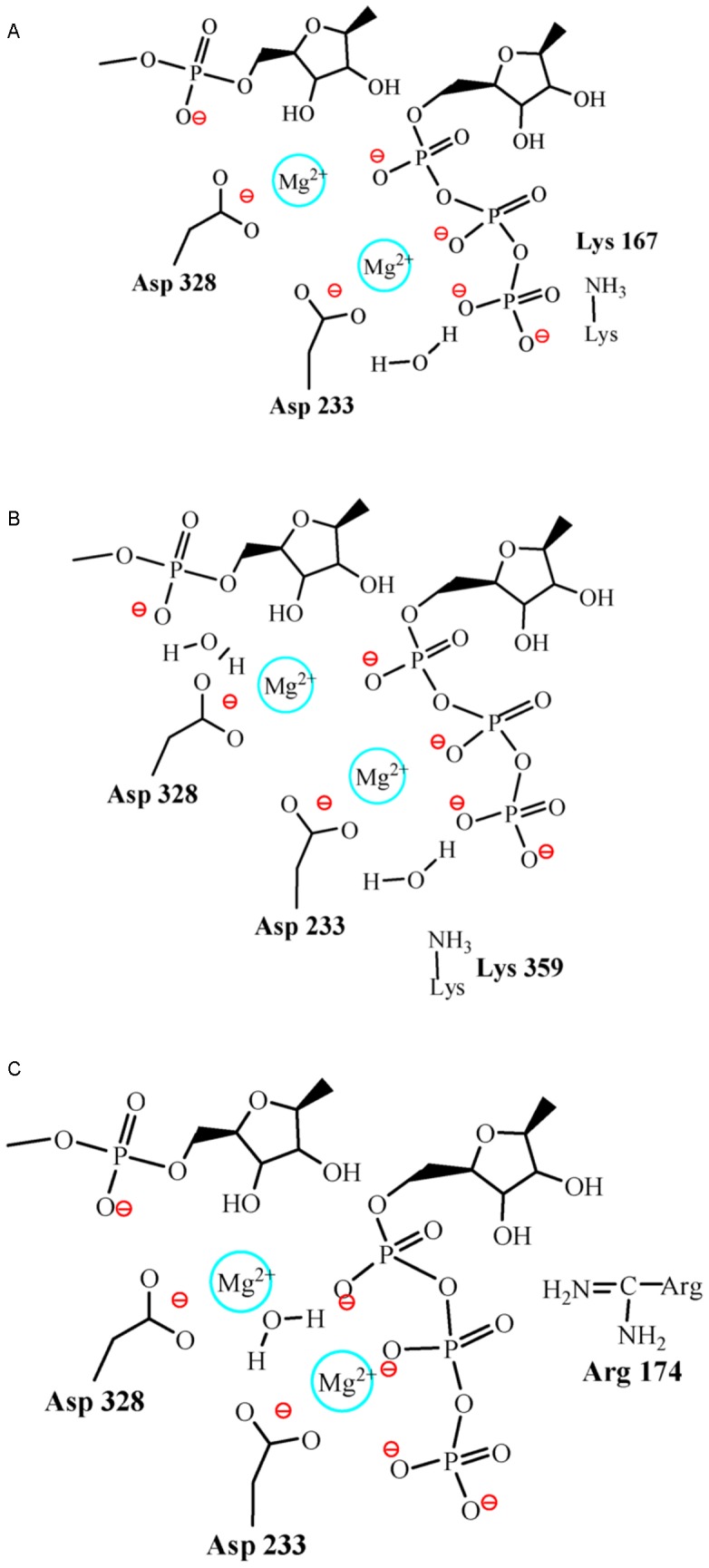
Three truncated systems, including Lys167 (A), Lys359 (B) and Arg174 (C) separately, were used for quantum mechanics (QM) calculations. Each system contains the phosphate and sugar of the primer terminus adenosine nucleotide, Asp328, Asp233, crystal water, two Mg^2+^ ions, rCTP molecule.

After MD simulations of the 3D^pol^-RNA-rCTP complex reached equilibration, the conformations sampled from five independent trajectories were used for the cluster analysis [46], in which a representative structure of the 3D^pol^-RNA-rCTP complex was selected from the largest cluster. In order to use QM calculation efficiently to explore the enzyme catalysis, the representative structure of the 3D^pol^-RNA-rCTP complex was truncated by removing components except for the residues Asp233, Asp328, Lys167, Lys359, Arg174, the sugar and phosphate of the primer terminus adenosine nucleotide, rCTP molecule, two metal ions (Mg^2+^) and a few waters close to the catalysis site. Considering computational cost, the truncated structure was split into three different structures including three residues Lys167, Lys359 and Arg174 separately (see their representations in Figure 2), and three structures were used for the following QM calculations carried out at the HF/6-31g(d) level. All the stationary points for the reactants, products, possible intermediates (IM) and transition states (TS) were located by performing geometry optimization with just fixing backbone atoms to their original positions, and their features (such as local minima or first-order saddle points) were identified by performing frequency calculations. All calculations were performed by using Gaussian 09 program package [47]. For all the cited energies, the zero-point energy corrections have been taken into account.

## Results/Discussion

### PV 3D^pol^ and RNA Form a Stable Complex

From the MD simulations of the PV 3D^pol^ apo and complex structures, the RMSD (Root Mean Square Deviation) and RG (radius of gyration) values were calculated for all backbone heavy atoms of PV 3D^pol^ against simulation time, and the RMSD and RG curves for PV 3D^pol^ in the apo and complex forms were plotted in Supporting Figures S3 and S4 respectively. From the results, one may expect that the PV 3D^pol^ should be more flexible in the apo form than in the complex form, but this expectation has to be confirmed by further analysis, such as the calculations of B-factors and principal component analysis (PCA), which will be discussed in the following paragraphs. In comparison with other four MD trajectories of the PV 3D^pol^ apo structure, the first MD trajectory demonstrated the large-amplitude fluctuation of RMSD values in the range of 13–15 ns. This should mainly be ascribed to the motions of the thumb and the segment (residues 210–220) of the palm, because the B-factor values of the segment (residues 210–220) of the palm and the thumb domain were in general higher than those from other four trajectories, see Supporting Figure S5. This could be confirmed by calculating the backbone heavy-atom RMSD values for the PV 3D^pol^ excluding two segments (residues 210–220, 380–461) (see the green curve in Supporting Figure S3). It has been shown, from the RG curves, that larger RG values were observed for PV 3D^pol^ in the complex form than in the apo form. The reason is simple: the protein core of PV 3D^pol^ has to be expanded in order to accommodate the entry of RNA. Among the five independent MD trajectories of PV 3D^pol^ in the apo form, two trajectories clearly showed the expansion of PV 3D^pol^, see Supporting Figure S4. Comparing to other four MD trajectories, the relatively large RG values of the PV 3D^pol^ apo form in the first MD trajectory may result in its instability during the MD simulation, explaining the large-amplitude RMSD fluctuation observed in the first MD trajectory (see Supporting Figure S3). When RNA double strands bound to the polymerase, the contraction and expansion of the polymerase were restricted due to the interactions between the polymerase and RNA strands [13]; we will discuss these findings in the following paragraphs.

**Figure 3:**
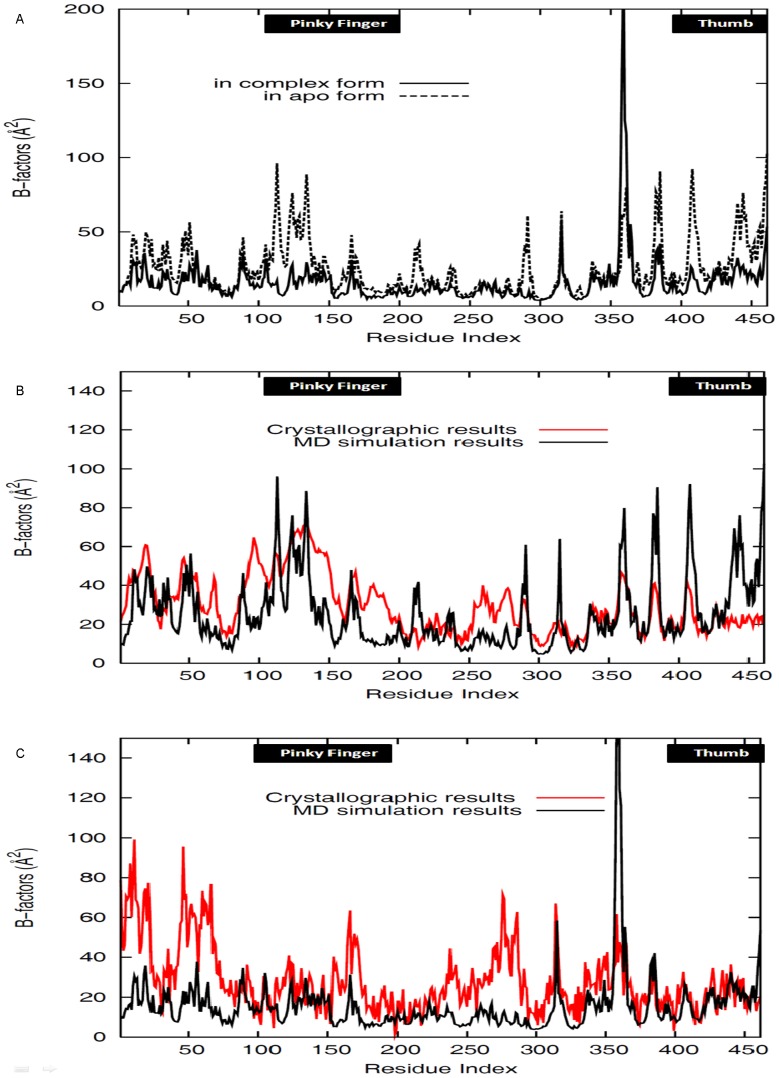
B-factor values of the backbone alpha carbons for PV 3D^pol^. (A) B-factor values of the backbone alpha carbons for PV 3D^pol^ in the apo and complex forms, which were obtained by averaging over five independent equilibrated MD trajectories. (B) B-factors of the backbone alpha carbons for PV 3D^pol^ in the apo form, which were obtained from MD simulation and crystallographic result. (C) B-factors of the backbone alpha carbons for PV 3D^pol^ in the complex form, which were obtained from MD simulation and crystallographic result. Please note that in the plots the crystallographic B-factors were shifted down in order to have clear comparison with MD derived values. The regions of the pinky finger and the thumb are indicated by the black boxes in the figures.

B-factors of the backbone alpha carbons for PV 3D^pol^ in the apo and complex forms were measured in this study and were presented in Figure 3a and Supporting Figure S5. These results confirmed that the apo structure of PV 3D^pol^ was more flexible than its complex structure, especially in some regions, such as the pinky finger (residues 96–190) and the thumb (residues 386–461). This may be attributed to the binding of RNA primer/template, which is responsible for stabilizing the polymerase [13]. In addition, B-factor values obtained from MD simulations and from crystallographic results have been compared for the two forms of PV 3D^pol^ respectively, given in Figures 3b and 3c. Both crystallographic and MD derived results reached the same agreement that the presence of RNA would decrease the flexibility of the pinky finger (residues 96–190) and the thumb (residues 381–461).

**Figure 4:**
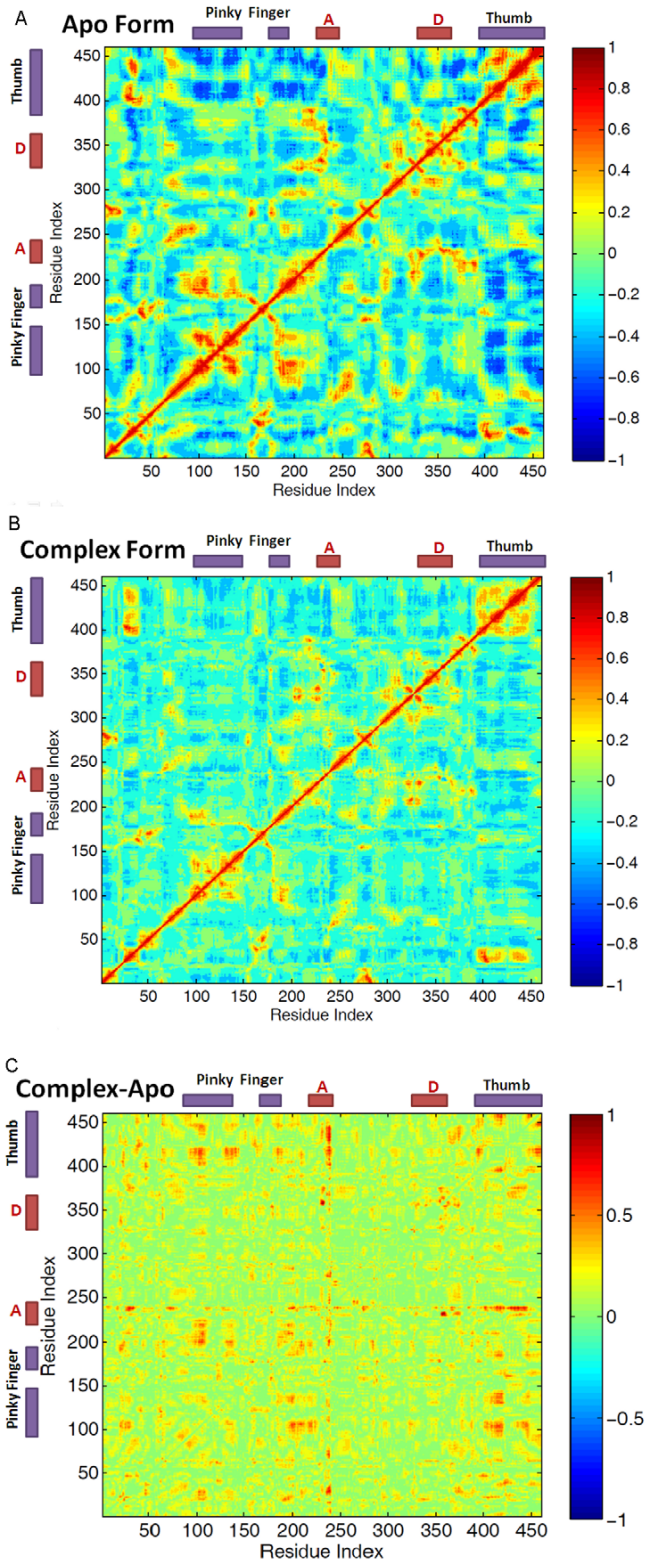
Dynamic cross correlation maps (DCCMs) for PV 3D^pol^ in the apo form (A) and the complex form (B). The results were obtained from five independent equilibrated MD trajectories, and (C) the absolute difference map, which were obtained by subtracting the DCCM of the apo form from that of the complex form. The purple boxes indicate the regions of the pinky finger and the thumb while the red boxes display the regions of the motifs A and D.

The dynamic cross correlation analysis were carried out on PV 3D^pol^ in the apo and complex forms respectively. As for the apo form of PV 3D^pol^, the negatively correlated motions of amino acid residues in PV 3D^pol^ were observed between the segment (residues 98–159) of the fingers and the segment (residues 408–461) of the thumb, shown in Figure 4a. It has been shown in the previous study [14] that the opening motion of the RNA polymerase was associated with the motions of the pinky finger and the thumb. This opening motion is necessary for the entrance of RNA into RNA polymerase core because the nascent RNA channel of the PV 3D^pol^ apo form is too small to fully accommodate RNA double strands. When RNA entered into polymerase core, the stable PV 3D^pol^-RNA complex was formed, such that the strong negatively correlated motions (the correlation value of −0.6 and less) of amino acid residues from the pinky finger and the thumb became less negatively correlated, which has been shown in the dynamic cross correlation map (DCCM) for PV 3D^pol^ in the complex form, given in Figures 4b. When we calculated the absolute difference between the two correlation maps of PV 3D^pol^ in the apo and complex forms, the significant difference was observed for the correlation between the pinky finger and the thumb, see Figure 4c. However, according to the dynamic cross correlation maps for PV 3D^pol^ in the apo and complex forms, strong positively correlated motions (the correlation value of 0.6 and above) existed between amino acid residues from the segment (residues 23–34) of the fingertips and the segment (residues 404–445) of the thumb in either the presence of RNA or the absence of RNA, indicating that the RNA binding to the polymerase had limited effect on the interaction between the fingertips and the thumb.

From Figure S6, one can see that some residues from the pinky finger (residues 96–149) and thumb subdomains (residues 386–461) have close contact to the RNA primer/template. On one side, the coil segments of the pinky finger inserts into the RNA major groove, and on the other side, an alpha helical segment of the thumb (residues 386–461) packs into the RNA minor groove. In order to gain more information about the interactions between the polymerase and the RNA primer/template, we performed hydrogen bonding analysis, and results were given in Table 1. In this table, we observed that the positively charged residues from the polymerase, such as lysine and arginine, contributed predominately to the binding of RNA in the polymerase core, which has recently been discussed in details [48]. One should note that the fingers and thumb domains are rich in positively charged residues, such as lysine and arginine, which can facilitate the binding of RNA. On the other hand, from the B-factors shown in Figure 3, one can see that the pinky finger and thumb domains of PV 3D^pol^ in the complex form demonstrate the flexibility to a certain degree; the flexibility of the pinky finger and thumb domains may be necessary for the RNA steady translocation one step at a time during each cycle of nucleotide incorporation. However, it is very expensive to carry out traditional MD simulations to study the RNA translocation.

**Table 1:** The hydrogen bonding occupancy for the residue pairs between RNA and polymerase, which were calculated by averaging over five independent MD trajectories of PV 3D^pol^ in the complex form.

Polymerase		RNA	Hydrogen Bonding occupancy
**Thumb**	Ser400	RA488	85.60%
	Arg456	RC472	70.15%
	Lys405	RG487	78.72%
	Arg415	RC472	59.17%
**Pinky Finger**	Lys127	RU467	86.90%
	Thr114	RU467	93.38%
	Ser115	RG466	94.00%
	Arg128	RC484	65.72%
	Arg128	RA483	78.65%
	Ser115	RG466	78.75%

**Figure 5:**
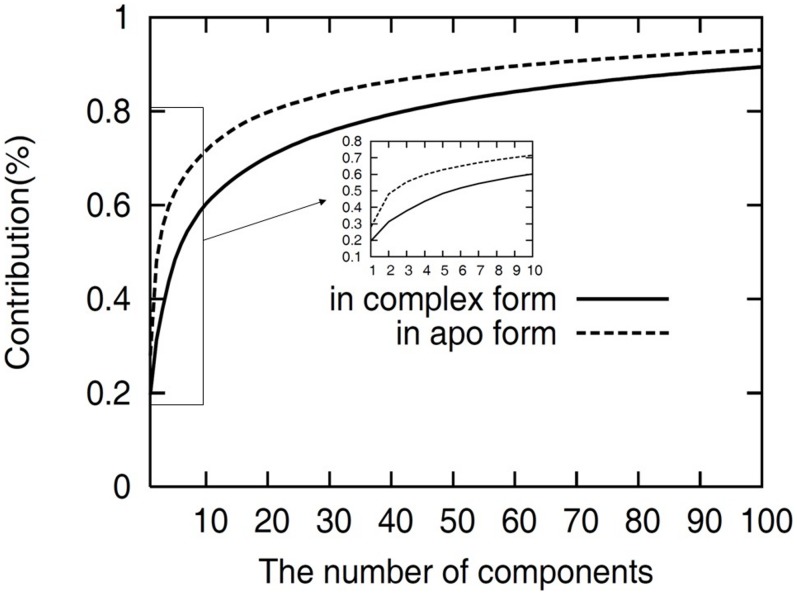
Accumulated contributions to the overall motion of PV 3D^pol^. The results were obtained from five independent equilibrated MD trajectories. The results of PV 3D^pol^ in the apo and complex forms were plotted against the number of principal components, indicated by the black dash and solid lines respectively.

To investigate the major contributions to the overall motion of PV 3D^pol^, we performed a principal component analysis (PCA) on the equilibrated MD trajectories of the simulation of the PV 3D^pol^ apo and complex structures. In the PCA, the first component (PC1) corresponds to the largest atomistic fluctuation around an average structure and the second component (PC2) represents the next largest atomic fluctuation, and so on. In Figure 5, we plotted the accumulated contributions to the overall motion of PV 3D^pol^ in the apo and complex forms against the number of principal components (PCs). From the figure, we observed that the accumulated contribution converges more quickly for PV 3D^pol^ in the apo form than in the complex form, such that over 70% of total fluctuation was contributed by the first 10 PCs for the apo form of PV 3D^pol^, but as for the 3D^pol^-RNA-rCTP complex the top 10 PCs only contributed the total motions by barely 60%. This indicates that the global dynamics of PV 3D^pol^ in the apo form can be described by a few low-frequency (large-magnitude) motions, and such large-magnitude motions are necessary for the entrance of NTP into RNA polymerase. In contrast, as the PV 3D^pol^ forms a complex with the RNA template/primer, more small-magnitude (high-frequency) motions contribute the overall motion of the complex, implying that the large conformational change in PV 3D^pol^ occurs more laboriously in the complex form than in the apo form.

**Figure 6:**
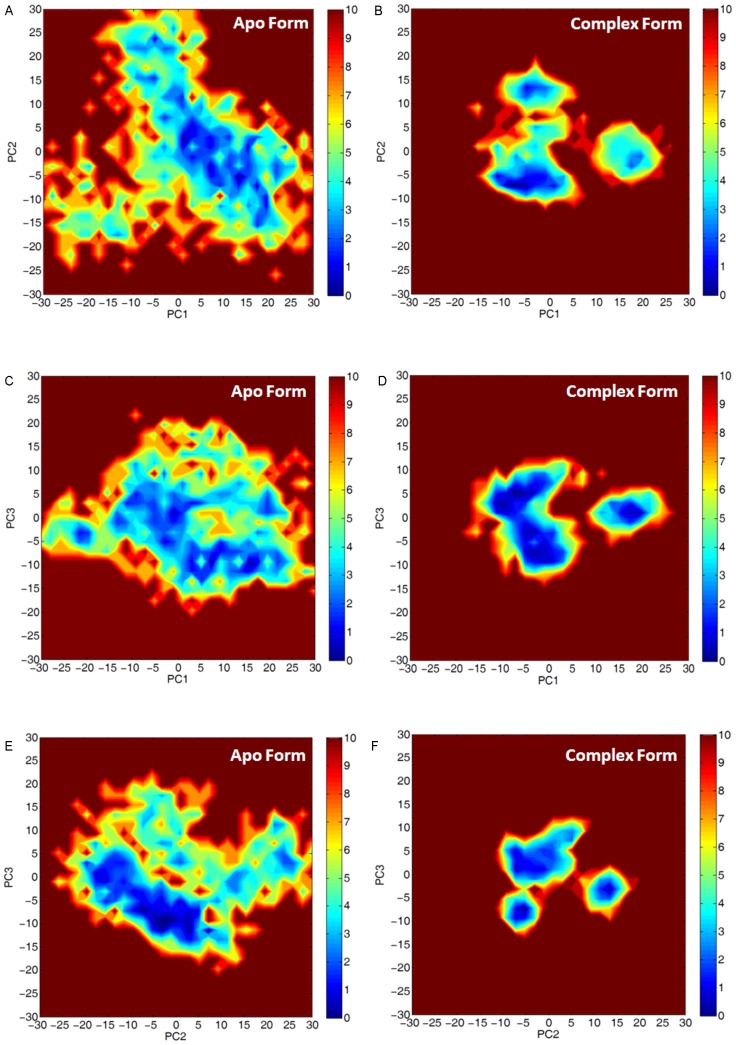
Free energy (in unit of kcal/mol) profiles of (A) 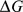
 **(PC1, PC2) for PV 3D^pol^ in the apo form, (B)** 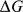 **(PC1, PC2) for PV 3D^pol^ in the complex form, (C)** 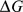 **(PC1, PC3) for PV 3D^pol^ in the apo form, (D)** 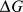 **(PC1, PC3) for PV 3D^pol^ in the complex form, (E)** 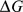 **(PC2, PC3) for PV 3D^pol^ in the apo form, (F)** 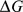 **(PC2, PC3) for PV 3D^pol^ in the complex form.** The first principal component (PC1) corresponds to the largest contribution, the second principal component (PC2) represents the next largest contribution to the overall motion of PV 3D^pol^, and so on.

Free energy landscapes in the space of three major principal components (PC1, PC2 and PC3) for PV 3D^pol^ in the apo and complex forms were plotted in Figure 6, illustrating that the free energy basins of PV 3D^pol^ were wider in the apo form than in the complex form. The calculations of 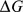
(PC1,PC2) for PV 3D^pol^ in the apo form, given in Figure 6a, have shown that the local energy minima spread out over a large portion of the free energy space. In contrast, the calculations of 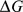
(PC1,PC2) for PV 3D^pol^ in the complex form, presented in Figure 6b, have shown three clear energy minima close to each other. This indicates that the motions of PV 3D^pol^ in the complex form are restricted in a narrow region and the large conformational changes of PV 3D^pol^ occur more easily in the apo form than in the complex form. The calculations of 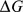
(PC1,PC3) and 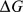
(PC2,PC3) for PV 3D^pol^ exhibited the similar behavior to that of 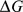
(PC1,PC2) in both the apo and complex forms, shown in Figure 6c–f.

In summary, our study suggested that the large conformational change of PV 3D^pol^ would occur more arduously in the presence of RNA than in the absence of RNA due to the interactions between RNA primer/template and the fingers on one side, and the interactions between RNA primer/template and the thumb on the other side. However, the apo and complex forms of PV 3D^pol^ both exhibited high flexibility at the motif-D loop containing the residue Lys359 (see Figure S5 and Figure 3), implying that the motif-D loop is the dynamic element independent of RNA binding. We also believe that there is incomplete information about what role the motif D is playing during nucleotide incorporation [14], [49]. In this study, we would like to explore the dynamic behaviors of motif D, in particular for the motif-D loop containing the residue Lys359, in order to understand the functions of motif D more completely.

### Does Lys359 of Motif D Serve as the Transporter of Incoming Nucleotide?

To the best of our knowledge, the dynamic elements of PV 3D^pol^, such as motifs A, D and F, should be essential to enzyme function. It is known that motif A plays an important role in enzyme catalysis, and it is clear that motif F is responsible for the binding of an incoming nucleotide as well as for the binding of RNA. Yang et al. [49] used solution-state nuclear magnetic resonance (NMR) to study PV 3D^pol^, and discovered that the motif D was unable to achieve its optimal position when an incorrect nucleotide bound to the RNA polymerase, implying that the conformational change of motif D could be the rate limiting step for nucleotide misincorporation.

**Figure 7:**
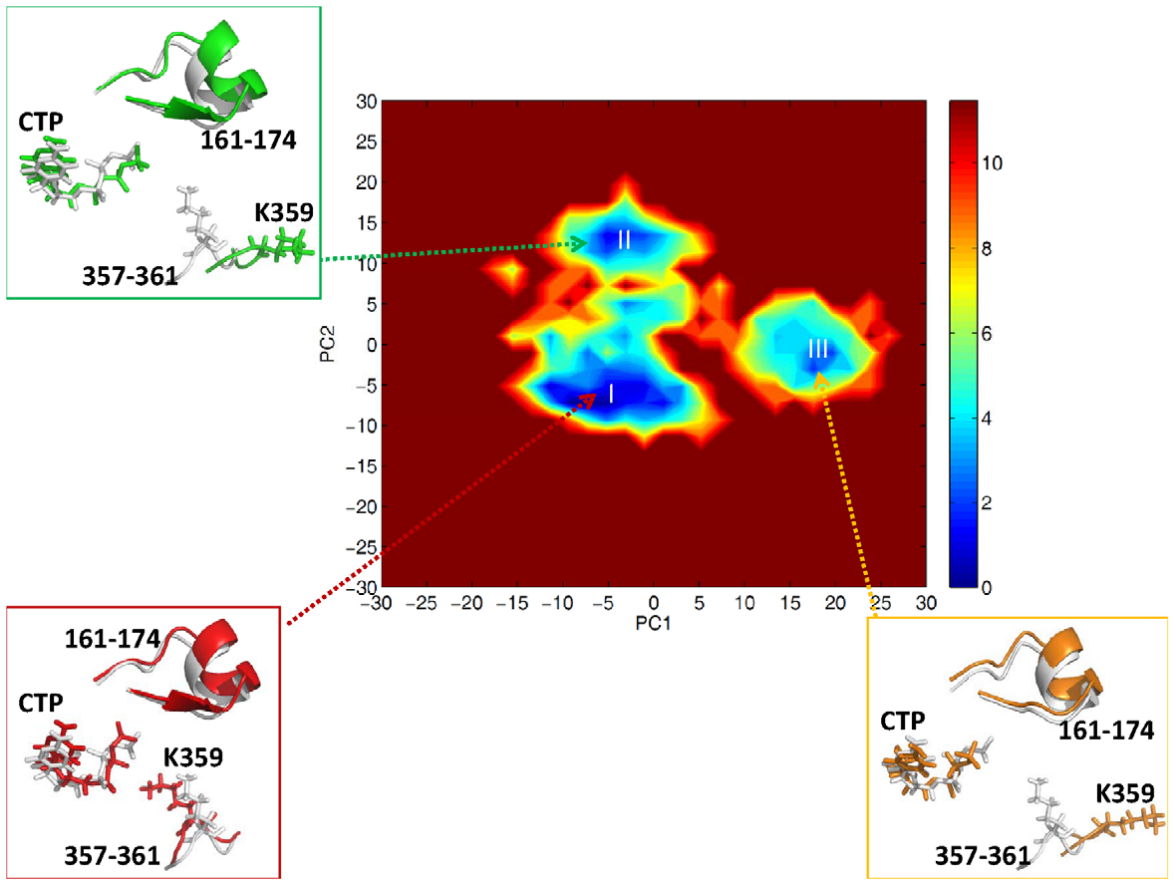
Free energy (in unit of kcal/mol) profile of 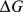
(PC1, PC2) for PV 3D^pol^ in the complex form. In the three states (I, II, III), the stick representations of CTP and the residues 357–361 of motif D, and the cartoon representation of the residues 161–174 of motif F, are depicted in red, green and orange respectively, and the crystal structure is indicated in white gray.

**Figure 8:**
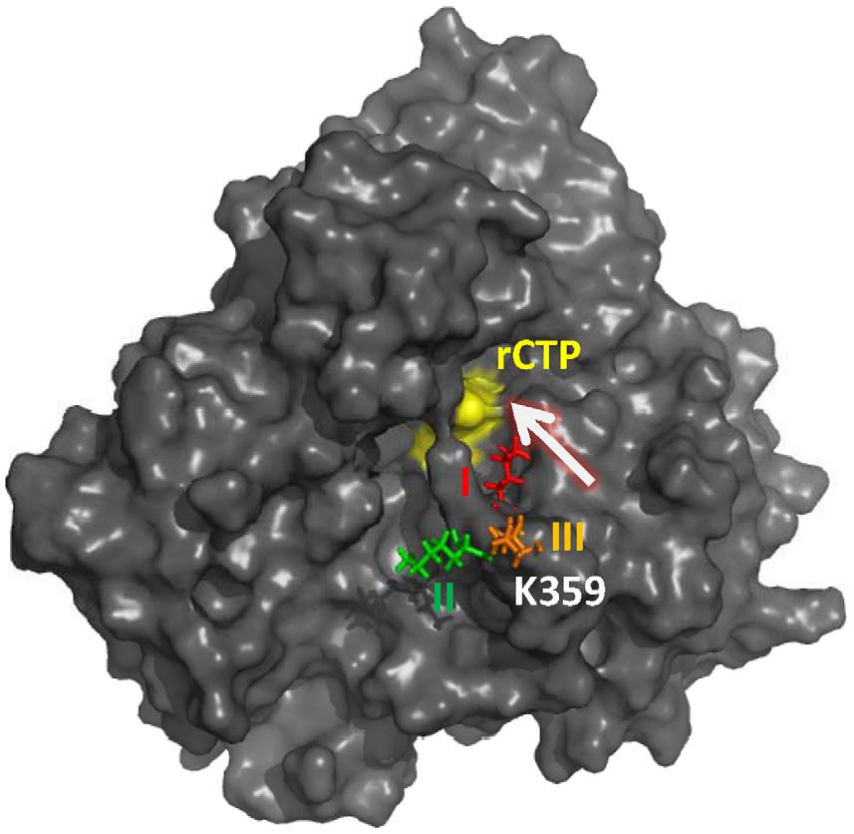
A surface representation (dark gray) of the 3D^pol^-RNA-rCTP complex structure. The entry pathway of rCTP molecule is indicated by a white arrow, and the residue Lys359 (stick representation) of motif D in three states (in the free energy landscape of 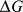
(PC1, PC2) given in Figure 7) is depicted in red (state I), green (state II) and orange (state III) respectively.

According to the free energy landscape of top two principal components (PC1 and PC2) for PV 3D^pol^ in the complex form, we observed three energy minima (I, II, III), see Figure 7. From the figure, we observed that, in the three states, the CTP molecule was positioned at the active site in the same way as in the static crystal structure, the segment (residues 161–174) of motif F was positioned slightly away from the active site, and the residue Lys359 of motif D exhibited higher mobility than other residues. In the state I, the side chain of Lys359 was oriented toward the active site, and the shortest distance was measured of 4.17 Å between the side chain atom NZ and an oxygen atom connecting to the β-phosphate of the incoming nucleotide. This indicates that it is possible for Lys359 to interact with the triphosphate moiety of the incoming nucleotide. However, in the states II and III, Lys359 moved far away from the active site. According to the transitions from either the states II or III to the state I, the side chain of Lys359 moved along the entry pathway of incoming nucleotide, see Figure 8, suggesting that one of the roles of Lys359 in nucleotide incorporation might facilitate the transportation of incoming nucleotide from outside to inside the polymerase PV 3D^pol^. Thus, in the future it would be interesting to investigate how the motif D influences the transportation of an incoming correct or incorrect nucleotide as well as the release of pyrophosphate.

### Interaction Between Motif D and Motif A should be Critical to the Polymerase Function

**Figure 9:**
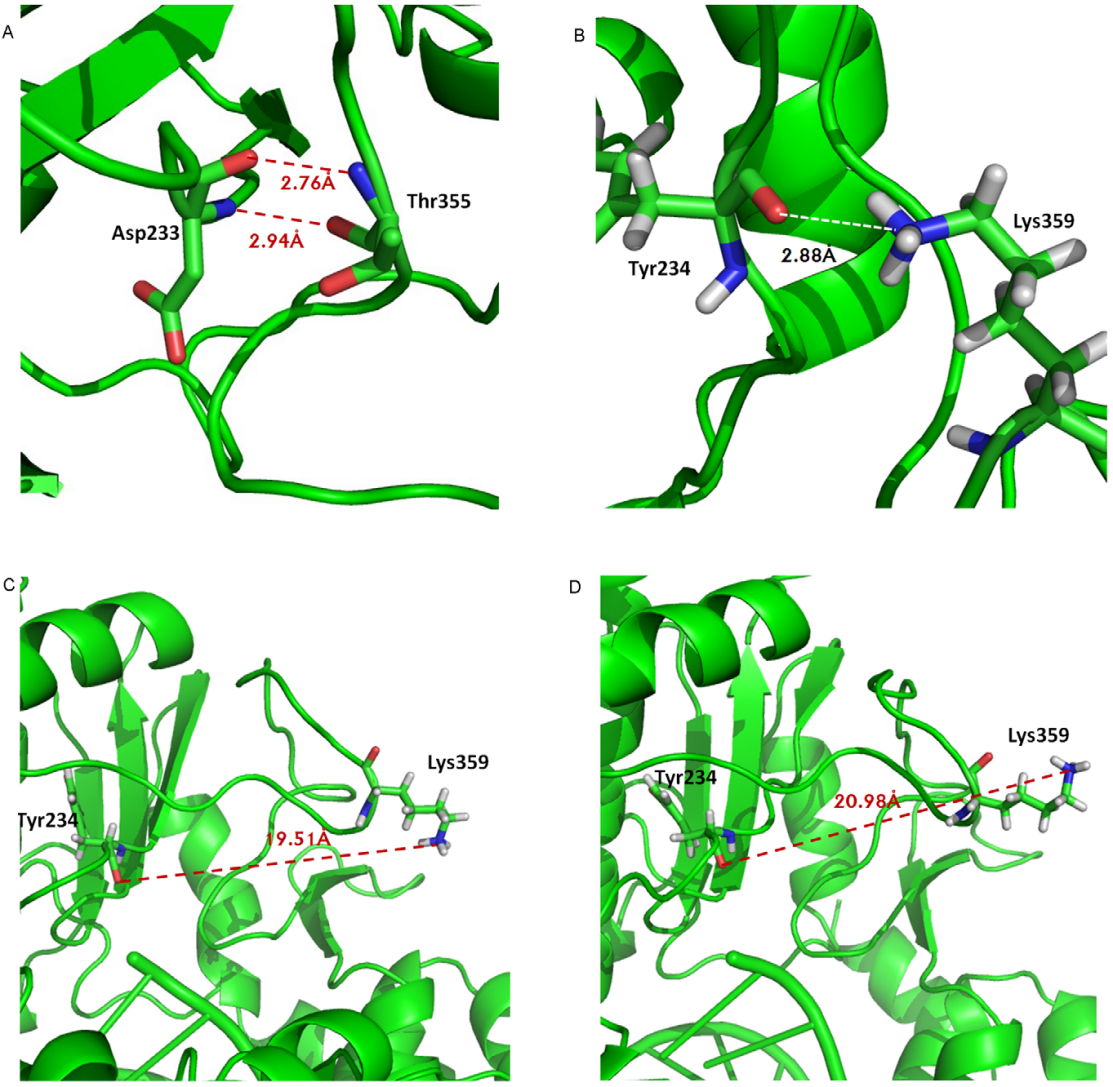
Free energy profile (in unit of kcal/mol) of 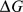
PC1, PC2) for PV 3D^pol^ in the complex form. (A) In the state I of the free energy landscape of 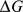
(PC1, PC2) given in Figure 7, the distances were measured between the backbone atoms of the residues Asp233 and Thr355. The distances were measured between the atom NZ of Lys359 and the atom O of Tyr234 in (B) the state I, (C) the state II, and (D) the state III, respectively.

From the dynamic cross correlation map for PV 3D^pol^ in the complex form, we observed the positively correlated motions (collective movement) between motif A (residues 229–240) and the segment (residues 354–362) of motif D, see Figure 4b. The collective movement between the two segments was mainly attributed by the interactions between the segment (residue 232–235) of motif A and the segment (residues 354–355) of motif D, see Table 2. Among them, the strongest positively correlated motions was found between Asp233 and Thr355 because two stable backbone hydrogen bonds were formed between the two residues (the hydrogen bonding occupancy of about 70% for each hydrogen bond), see Figure 9a. In addition, the third hydrogen bond (the hydrogen bonding occupancy of 21.07%) existed between the side chain atoms of the residues Asp233 and Thr355. The collective movement between motifs A and D, was also contributed by the hydrogen bonds formed between the residues pairs, such as Phe232/Thr355, Asp233/Met354, Asp234/Met355, and Tyr234/Thr355. Our MD results were consistent with the experimental observation by Peersen [13], showing the significant collective movement between motifs A and D.

**Table 2:** The correlation values of 0.6 and above were measured between the residue pairs from motifs A and D, based on the DCCM of PV 3D^pol^ in the complex form.

Motif A	Motif D	Correlation
Phe232	Thr355	0.632
Asp233	Met354	0.676
Asp233	Thr355	0.754
Tyr234	Met354	0.698
Tyr234	Thr355	0.688

Based on the free energy landscape of top two principal components (PC1, PC2) for PV 3D^pol^ in the complex form, we further examined the motions of motif A (residues 229–240) and the segment (residues 354–362) of motif D in three preferential states (I, II, III), see Figure S7. As we mentioned earlier, in the state I, the segment (residues 354–362) of motif D was poised toward the active site, however, in the states II and III, the segment (residues 357–362) of motif D moved away from the active site. Similarly, the motif A exhibited the same behavior because of the strong interactions between the motifs A and D. In addition, our results are in agreement with the experimental finding indicating that the closed and open states of the RdRp active site should be associated with the arrangement of motifs A and D [13]. For instance, the state I, displaying that the motifs A and D were poised toward the active site, reflected the closed state of the active site. In contrast, both the motifs A and D moved away from the active site in the states II and III, which reflected the open states of the active site.

**Figure 10:**
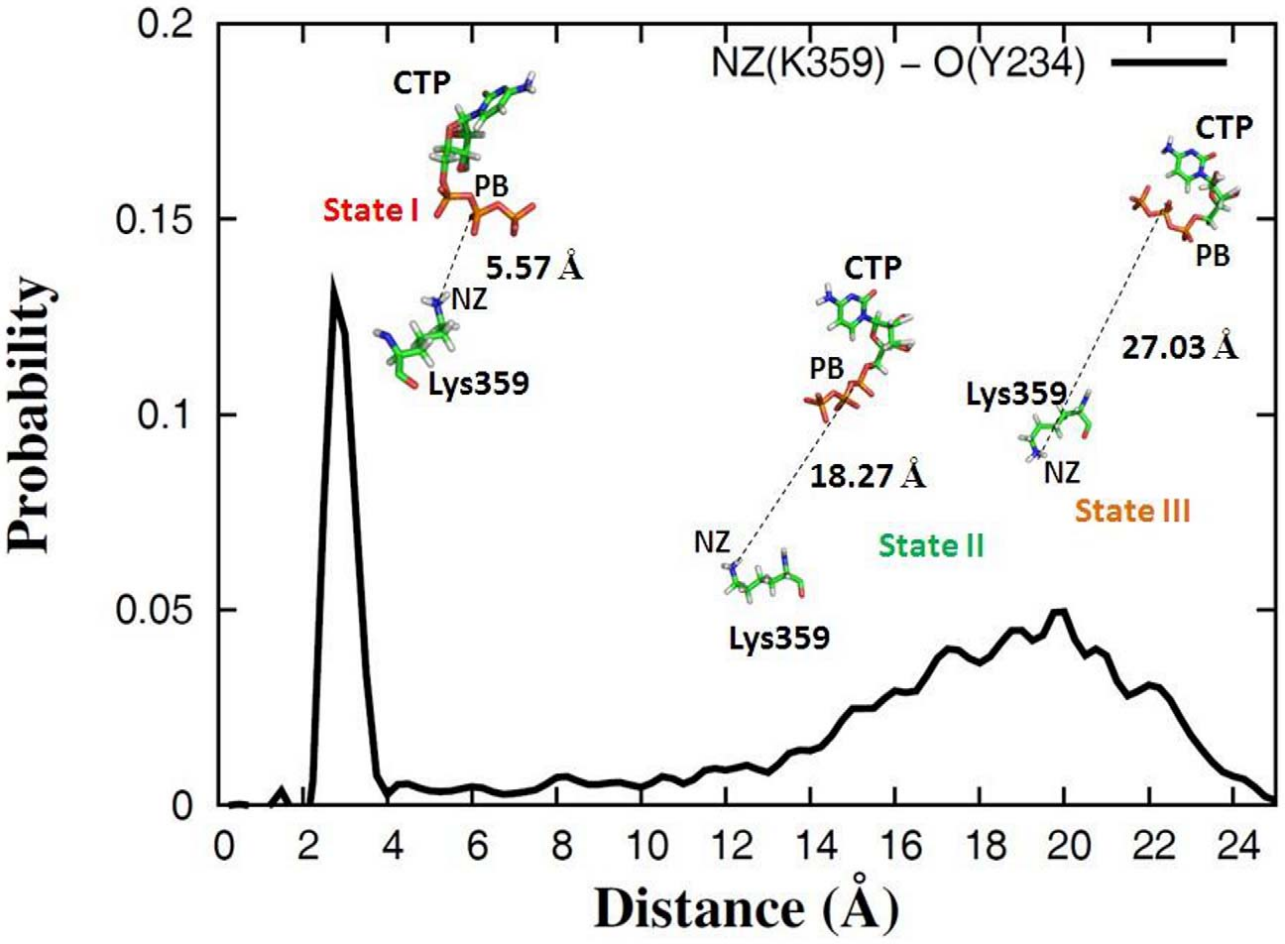
The distribution of the distance between the side chain atom NZ of Lys359 and the backbone atom O of Tyr234. The results were calculated from five independent trajectories of 3D^pol^-RNA-rCTP complex structure. In the plot, the three preferential states (I, II, II) in the free energy landscape of 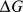
(PC1, PC2) are labeled, and their representative conformations associated with Lys359 and rCTP are shown as well. In addition, the distances were measured between the PA (γ-phosphate) atom of rCTP and the side chain atom NZ of Lys359 in the three states.

To gain more information about the dynamics of Lys359, we calculated the distribution of the distance between the side chain atom NZ of Lys359 and the backbone atom O of Thr234 from five independent MD trajectories, which was plotted in Figure 10. As we mentioned above, in the state I, a hydrogen bond could exist between Lys359 and Thr234, see Figure 9b. However, comparing to Thr234, the side chain of Lys359 was much more flexible and moved away from the active site in the states II or III, resulting in the increased distance between the atom NZ of Lys359 and the atom O of Thr234, shown in Figures 9c and 9d. In addition, for each state, the distance was measured between the β-phosphate atom (PB) of rCTP and the side chain atom NZ of Lys359 (see Figure 10), indicating that the motif-D loop may play a role in transporting incoming nucleotide.

In summary, our MD study of PV 3D^pol^ was consistent with the experimental observation by Peersen [13], showing that the movement of motif A is the key element forming the active site for enzyme catalysis. The dynamic element, motif D, has great impact on the motions of motif A through a number of hydrogen bonds formed between the residues from motifs A and D. It is undoubted that, in addition to the conformational changes of motif D, the interplay between motif A and motif D should be critical to enzyme catalysis.

### Does Lys359 from Motif D Involve in the Nucleotidyl Transfer?

As we mentioned earlier, two-metal ions were involved in the chemistry step of the nucleotide incorporation catalyzed by PV 3D^pol^ [32], [33], and two proton transfers were observed in the nucleotidyl transfer catalyzed by RNA or DNA polymerases [34]. However, a few questions need to be addressed: Which amino acid residue serving as a general acid donates a proton to the pyrophosphate (PPi) leaving group? Is it from motif D [35] or motif F [13]? If Lys359 serves as the general acid, how does it donate the proton to the PPi leaving group? Where is the proton from the primer terminus 3′-OH transferred to?

**Figure 11:**
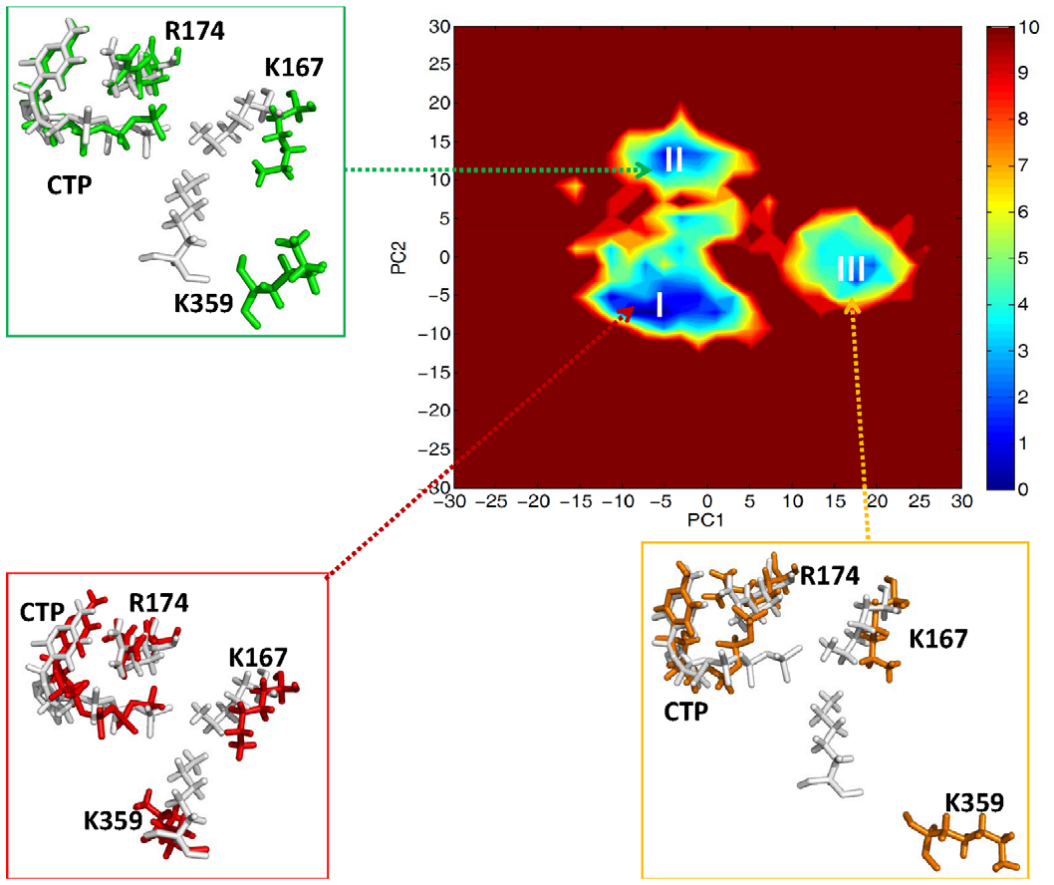
Free energy (in unit of kcal/mol) profile of 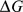
(PC1, PC2) for PV 3D^pol^ in the complex form. In the three states (I, II, III), the stick representations of CTP, Arg163, Lys167 and Arg174, are depicted in red, green and orange respectively, and the crystal structure is indicated in white gray.

To explore which residue of PV 3D^pol^ could act as the proton donor in nucleotidyl transfer, we examined some acidic residues close to the active site of PV 3D^pol^, such as Lys359, Lys167, and Arg174. Available crystal structures [13] of PV 3D^pol^ in different kinetic states showed that Arg174 instead of Lys359 was poised for enzyme catalysis. According to the PCA result of the 3D^pol^-RNA-rCTP complex presented in Figure 7, Lys359 stayed close to the active site in the state I due to the hydrogen bond formed between the side chain atom NZ of Lys359 and the backbone atom O of Thr234. This may provide the possibility for Lys359 to donate a proton to rCTP. Since a few waters have been found between Lys359 and rCTP, it is possible for Lys359 to indirectly donate the proton to leaving PPi group through waters if Lys359 serves as the general acid in enzyme catalysis. As for Lys167, it demonstrated moderate flexibility, but we observed that it always stayed away from the active site in three different conformational states, see Figure 11. Since the strong positively correlated motion (the correlation value of 0.6 and above) was observed between the residue Lys167 and the index finger (see Figure 4b), the movement of Lys167 should be affected by the index finger, which was observed moving away from the active site from current MD simulations. Thus, Lys167 has low possibility serving as a candidate for the proton donor in nucleotidyl transfer. From either crystal structures or conformations sampled from MD simulations, we observed that the residue Arg174 kept close to NTP. In addition, a few studies of other enzymes have demonstrated that arginine was able to involve in the enzyme catalysis through lowing pKa of arginine [50], [51]. Nevertheless, based on the pH titration curves in the kinetics experiments of PV 3D^pol^, Cameron et al [35] believed that Lys359 instead of Arg174 or Lys167 would likely be the good candidate. In order to gain insight into the mechanism of the proton transfer, we carried out QM calculations on the 3D^pol^-RNA–rCTP complex.

**Figure 12:**
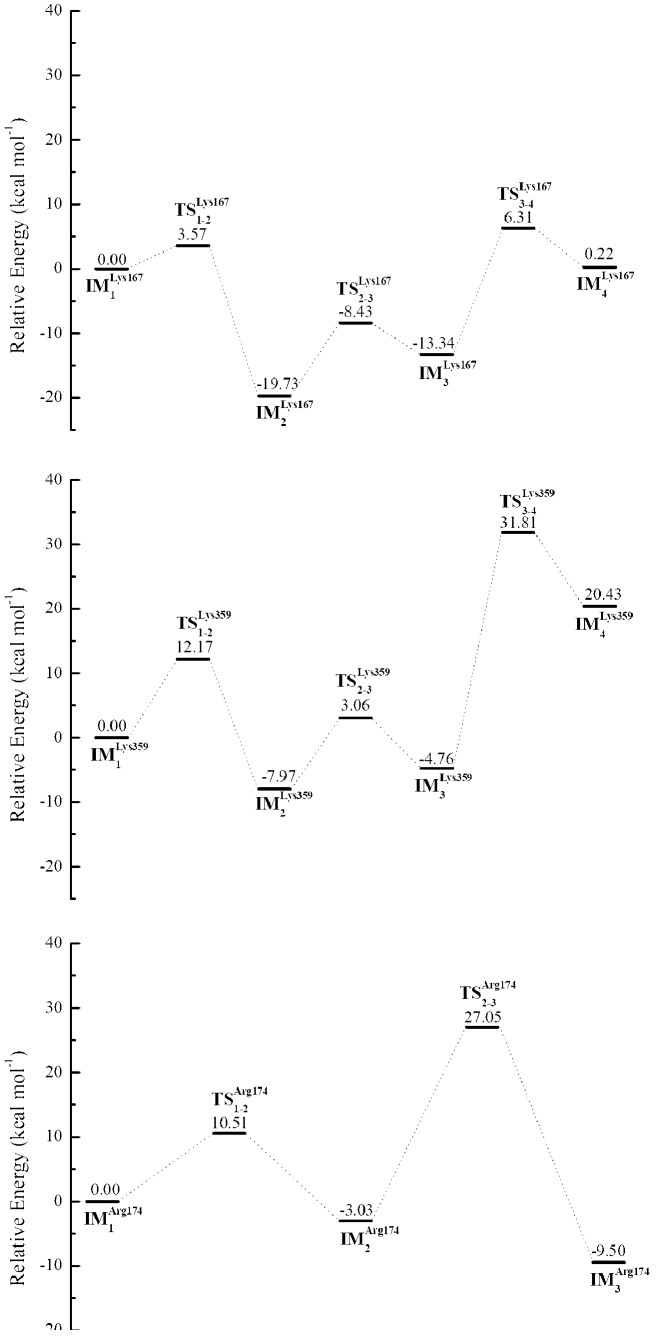
Relative Energy profiles were summarized from the quantum mechanics (QM) calculations of three truncated systems with Lys167 (A), Lys359 (B) and Arg174 (C). IM and TS represent intermediate and transition states respectively. The subscript of IM indicates the number of a state, and the subscript of TS represents the transition from one state to another. Different IM and TS states were given in the Figure S8 through Figure S10 of Supporting Information.

Considering the computational cost of QM calculations carried out on the 3D^pol^-RNA–rCTP complex, a representative structure of 3D^pol^-RNA–rCTP complex was obtained in the largest cluster of conformations sampled from MD simulations, and then it was truncated by removing its components except for the residues Asp233, Asp328, Lys167, Lys359, Arg174, the primer terminus phosphate and sugar of RNA primer, the rCTP molecule, two metal ions and a few waters near the active site. In order to explore the role of each acidic residue (Lys167, Lys359 and Arg174), as well as to save the computational expanse, the truncated structure was split into three different structures including Lys167, Lys359 and Arg174 separately, see Figure 2. The three structures were used for QM calculations at the HF/6-31g(d) level respectively, and their energy profiles were summarized in Figure 12. For all intermediate (IM) and transition states (TS), their representations were given in Figures S8, S9, S10.

From the QM calculations of the truncated structure with Lys167, the energy barrier of about 3 kcal/mol was calculated for the proton transfer from Lys167 to one of oxygen atoms connecting to the γ-phosphate of the incoming nucleotide, and a water molecule played an important role in this process, shown in Figure 12a and Figure S10. Moreover, the intermediate state 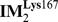
(see Figure S8c) with the protonated rCTP was much more stable than the reactant 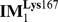
(see Figure S8a). As for the transition from the intermediate state 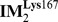 to the intermediate 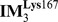
(see Figure S8e) through the transition state 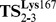
(see Figure S8d), the energy barrier was about 11 kcal/mol (see Figure 12a). However, when the un-protonated primer terminus 3′-O attacked the α-phosphate of the incoming nucleotide rCTP (see 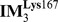 and 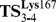 of Figure S8), the energy barrier of about 20 kcal/mol was calculated for this step, and the product 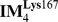
(see Figure S8g) was not stable thermodynamically (see Figure 12a). This indicates that in the presence of Lys167 it is more difficult for the chemical bond formation between the un-protonated primer terminus 3′-O and the α-phosphate of the incoming nucleotide than for two proton transfers to occur (the primer terminus 3′-OH donated a proton to its neighboring backbone phosphate of the primer, and Lys167 donated a proton to the incoming nucleotide).

As for the truncated structure with Lys359, the energy barrier of about 12 kcal/mol was measured for the proton transfer from Lys359 to the incoming nucleotide rCTP. As for the proton transfer from the primer terminus 3′-OH to its neighboring backbone phosphate of the primer, the energy barrier was found to be around 12 kcal/mol. However, these two energy barriers were much lower than the energy barrier (about 35 kcal/mol) for the bond formation between the un-protonated terminus 3′-O (O3′) and the α-phosphate (PA) of the incoming nucleotide, see Figure 12b and Figure S9. This is similar to the case of Lys167. According to the crystal structures of PV 3D^pol^ in different kinetic states, Lys359 was not poised for the enzyme catalysis [13]. However, the kinetic studies of the mutations at Lys359 from Cameron lab [34] have shown that Lys359 should be the good candidate for the general acid involving in the enzyme catalysis. In addition, the computational study of HIV-reverse transcriptase [52] showed that Lys220 was able to move the active site and to serve as the general acid in the nucleotidyl transfer. Therefore, it was possible for Lys359 indirectly donate the proton to the incoming nucleotide in the case of PV 3D^pol^.

From the QM calculations of the system with Arg174, it was more difficult for the un-protonated primer terminus 3′-O to form a chemical bond with the α-phosphate of the incoming nucleotide (energy barrier of about 30 kcal/mol) than for the proton transfer (energy barrier of about 10 kcal/mol) to occur between the primer terminus 3′-OH and Asp233 through a water molecule, see Figure 12c and Figure S10. From the transition state 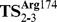
(see Figure S10d), we observed that Arg174 did not donate a proton to the PPi leaving group but it just had a hydrogen bonding interaction with the leaving PPi group in the reaction. This study suggested that it was more difficult for an arginine to donate a proton to incoming nucleotide than for a lysine to do. Nevertheless, in the case of PV 3D^pol^ Arg174 might facilitate enzyme catalysis through the hydrogen bonding interaction with the incoming nucleotide.

In summary, based on above QM calculation results, the kinetic studies of PV 3D^pol^ done by Cameron group [34] and the crystal structures of PV 3D^pol^ in different EC states solved by Peersen group [13], we provided our understanding about the enzyme catalysis in PV 3D^pol^. Firstly, the proton of the primer terminus 3′-OH may be donated to its neighboring backbone phosphate of the primer, and the bond formation between the atoms PA and O3′ could be the rate limiting step in the chemistry. Secondly, Arg174 should facilitate enzyme catalysis through the hydrogen bonding interaction with the incoming nucleotide. Thirdly, Lys359 may indirectly donate a proton to the PPi leaving group through some intermediates (such as waters). Fourthly, comparing to Arg174 and Lys359, the possibility for Lys167 to serve as the general acid is low. In addition, as we mentioned earlier, before the chemistry step of nucleotide incorporation, Lys359 may serve as the transporter of incoming nucleotide. Here, after the chemistry step, Lys359 again may serve as the transporter of outgoing PPi group.

## Supporting Information

Figure S1 **A front view (A) and a top view (B) of the cartoon representation of ligand-free PV 3D^pol^ (PDB code: 1RA6).** Three domains of PV 3D^pol^, fingers, palm and thumb, are depicted in blue, green and red, respectively.
(TIFF)

Figure S2 **Nucleotide sequences of the RNA primer and template.** Nucleotide sequences (A) of the RNA primer and template (PDB code: 3OL7) with the cytosine nucleotide (in red) that is to be deleted. The crystal structure (PDB code: 3OL7) reflects a state of the post-chemistry step of the nucleotide incorporation. After deleting the cytosine nucleotide from the RNA sequence in (A), the final nucleotide sequences (B) of the RNA primer and template were retained in the structure into which rCTP molecule was built.
(TIFF)

Figure S3 **Backbone heavy-atom RMSD curves of PV 3D^pol^.** The results were calculated from five independent MD simulations of the PV 3D^pol^ apo (red) and complex (black) structures. On the top-box, the backbone heavy- atom RMSD values were calculated from the first MD simulation for the apo form of PV 3D^pol^ excluding two segments (residues 210–220 and 380–461) and were plotted against simulation time (green curve).
(TIFF)

Figure S4 **Radius of gyration (RG) curves for the backbone heavy atoms of PV 3D^pol^ in the apo (red) and complex (black) forms.** The results were calculated from five independent MD simulations of the PV 3D^pol^ apo and complex structures.
(TIFF)

Figure S5 **B-factors of the backbone alpha carbons for PV 3D^pol^ in the apo (red) and complex (black) forms.** The results were obtained from five independent MD simulations of the PV 3D^pol^ apo and complex structures respectively.
(TIFF)

Figure S6 **A top view (A) and a side view (B) of the 3D^pol^-RNA-rCTP complex structure with the cartoon representation of protein (green) and VDW representation of RNA (white).** Segments from the pinky finger and the thumb domain are indicated in blue and red respectively.
(TIFF)

Figure S7
**Free energy (in unit of kcal/mol) profile of** 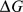 **(PC1, PC2) for PV 3D^pol^ in the complex form.** In the three states (I, II, III), the stick representation of CTP, and the cartoon representation of motif A and residues 354–362 of motif D, are depicted in red, green and orange respectively, and the crystal structure is indicated in white gray.(TIFF)

Figure S8
**Illustration of the reactant** 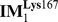 **(A), the transition state** 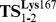 **(B), the intermediate state** 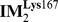 **(C), the transition state** 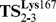 **(D), the intermediate state** 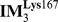 **(E), the transition state** 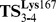 **(F), and the product** 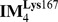 **(G) in the nucleotidyl transfer.** The results were obtained from the quantum mechanics calculations carried out on the truncated system with Lys167. The subscript of the intermediate state (IM) indicates the number of a state, and the subscript of the transition state (TS) represents the transition from one state to another.(TIFF)

Figure S9
**Illustration of the reactant** 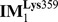 **(A), the transition state** 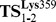 **(B), the intermediate state** 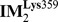 **(C), the transition state** 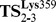 **(D), the intermediate state** 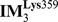 **(E), the transition state** 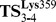 **(F), and the product** 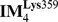 **(G) in the nucleotidyl transfer.** The results were obtained from the quantum mechanics calculations carried out on the truncated system with Lys359. The subscript of the intermediate state (IM) indicates the number of a state, and the subscript of the transition state (TS) represents the transition from one state to another.(TIFF)

Figure S10
**Illustration of the reactant** 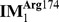 **(A), the transition state** 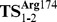 **(B), the intermediate state** 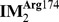 **(C), the transition state** 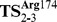 **(D), and the product** 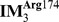 **(E) in the nucleotidyl transfer.** The results were obtained from the quantum mechanics calculations carried out on the truncated system with Arg174. The subscript of the intermediate state (IM) indicates the number of a state, and the subscript of the transition state (TS) represents the transition from one state to another.(TIFF)

## References

[1] Morrow CD, Warren B, Lentz MR (1987) Expression of enzymatically active poliovirus RNA-dependent RNA polymerase in Escherichia coli. Proc Natl Acad Sci USA 84: 6050–6054. 2819863 10.1073/pnas.84.17.6050 PMC299005

[2] Thompson AA, Peersen OB (2004) Structural basis for proteolysis-dependent activation of the poliovirus RNA-dependent RNA polymerase. EMBO J 23: 3462–3471. 15306852 10.1038/sj.emboj.7600357 PMC516629

[3] Love RA, Maegley KA, Yu X, Ferre RA, Lingardo LK, et al (2004) The Crystal Structure of the RNA-Dependent RNA Polymerase from Human Rhinovirus: A Dual-Function Target for Common Cold Antiviral Therapy. Structure 12: 1533–1544. 15296746 10.1016/j.str.2004.05.024

[4] Ferrer-Orta C, Arias A, Perez-Luque R, Escarmís C, Domingo E, et al (2004) Structure of Foot-and-Mouth Disease Virus RNA-dependent RNA Polymerase and Its Complex with a Template-Primer RNA. J Biol Chem 279: 47212–47221. 15294895 10.1074/jbc.M405465200

[5] Hansen JL, Long AM, Schultz SC (1997) Structure of the RNA-dependent RNA polymerase of Poliovirus. Structure 5: 1109–1122. 9309225 10.1016/s0969-2126(97)00261-x

[6] Crotty S, Cameron CE, Andino R (2001) RNA virus error catastrophe: Direct molecular test by using ribavirin. Proc Natl Acad Sci USA 98: 6895–6900. 11371613 10.1073/pnas.111085598 PMC34449

[7] Graci JD, Cameron CE (2006) Mechanisms of action of ribavirin against distinct viruses. Rev Med Virol 16: 37–48. 16287208 10.1002/rmv.483 PMC7169142

[8] Graci JD, Harki DA, Korneeva VS, Edathil JP, Too K, et al (2007) Lethal mutagenesis of poliovirus mediated by a mutagenic pyrimidine analogue. J Virol 81: 11256–11266. 17686844 10.1128/JVI.01028-07 PMC2045539

[9] Campagnola G, Gong P, Peersen OB (2011) High-throughput screening identification of poliovirus RNA-dependent RNA polymerase inhibitors. Antiviral Res 91: 241–251. 21722674 10.1016/j.antiviral.2011.06.006 PMC3159743

[10] Bruenn JA (1991) Relationships among the positive strand and double-strand RNA viruses as viewed through their RNA-dependent RNA polymerases. Nucleic Acids Res 19: 217–226. 2014162 10.1093/nar/19.2.217 PMC333583

[11] Castro C, Arnold JJ, Cameron CE (2005) Incorporation fidelity of the viral RNA-dependent RNA polymerase: a kinetic, thermodynamic and structural perspective. Virus Res 107: 141–149. 15649560 10.1016/j.virusres.2004.11.004 PMC7125856

[12] Arnold JJ, Vignuzzi M, Stone JK, Andino R, Cameron CE (2005) Remote site control of an active site fidelity checkpoint in a viral RNA-dependent RNA polymerase. J Biol Chem 280: 25706–25716. 15878882 10.1074/jbc.M503444200 PMC1557591

[13] Gong P, Peersen OB (2010) Structural basis for active site closure by the poliovirus RNA-dependent RNA polymerase. Proc Natl Acad Sci USA 107: 22505–22510. 21148772 10.1073/pnas.1007626107 PMC3012486

[14] Moustafa IM, Shen H, Morton B, Colina CM, Cameron CE (2011) Molecular Dynamics Simulations of Viral RNA Polymerases Link Conserved and Correlated Motions of Functional Elements to Fidelity. J Mol Biol 410: 159–181. 21575642 10.1016/j.jmb.2011.04.078 PMC3114172

[15] Arnold JJ, Cameron CE (2000) Poliovirus RNA-dependent RNA Polymerase (3Dpol.) Assembly of stable, elongation-competent complexes by using a symmetrical primer-template substrate (sym/sub). J Biol Chem 275: 5329–5339. 10681506 10.1074/jbc.275.8.5329

[16] Arnold JJ, Cameron CE (2004) Poliovirus RNA-Dependent RNA Polymerase (3D^pol^): Pre-Steady-State Kinetic Analysis of Ribonucleotide Incorporation in the Presence of Mg2+. Biochemistry 43: 5126–5137. 15122878 10.1021/bi035212y PMC2426923

[17] Arnold JJ, Gohara DW, Cameron CE (2004) Poliovirus RNA-Dependent RNA Polymerase (3D^pol^): Pre-Steady-State Kinetic Analysis of Ribonucleotide Incorporation in the Presence of Mn2+. Biochemistry 43: 5138–5148. 15122879 10.1021/bi035213q PMC2426922

[18] Gohara DW, Arnold JJ, Cameron CE (2004) Poliovirus RNA-Dependent RNA Polymerase (3D^pol^): Kinetic, Thermodynamic, and Structural Analysis of Ribonucleotide Selection. Biochemistry 43: 5149–5158. 15122880 10.1021/bi035429s PMC2426919

[19] Pfeiffer JK, Kirkegaard K (2003) A single mutation in poliovirus RNA-dependent RNA polymerase confers resistance to mutagenic nucleotide analogs via increased fidelity. Proc Natl Acad Sci USA 100: 7289–7294. 12754380 10.1073/pnas.1232294100 PMC165868

[20] Vignuzzi M, Stone JK, Arnold JJ, Cameron CE, Andino R (2006) Quasispecies diversity determines pathogenesis through cooperative interactions in a viral population. Nature 439: 344–348. 16327776 10.1038/nature04388 PMC1569948

[21] Korneeva VS, Cameron CE (2007) Structure-function relationships of the viral RNA-dependent RNA polymerase: fidelity, replication speed, and initiation mechanism determined by a residue in the ribose-binding pocket. J Biol Chem 282: 16135–16145. 17400557 10.1074/jbc.M610090200 PMC2116994

[22] Kuchta RD, Mizrahi V, Benkovic PA, Johnson KA, Benkovic SJ (1987) Kinetic mechanism of DNA polymerase I (Klenow). Biochemistry 26: 8410–8417. 3327522 10.1021/bi00399a057

[23] Eger BT, Kuchta RD, Carroll SS, Benkovic PA, Dahlberg ME, et al (1991) Mechanism of DNA replication fidelity for three mutants of DNA polymerase I: Klenow fragment KF (exo+), KF (polA5), and KF (exo−). Biochemistry 30: 1441–1448. 1991125 10.1021/bi00219a039

[24] Zinnen S, Hsieh JC, Modrich P (1994) Misincorporation and mispaired primer extension by human immunodeficiency virus reverse transcriptase. J Biol Chem 269: 24195–24202. 7523369

[25] Tsai YC, Johnson KA (2006) A new paradigm for DNA polymerase specificity. Biochemistry 45: 9675–9687. 16893169 10.1021/bi060993z PMC7526746

[26] Dunlap CA, Tsai MD (2002) Use of 2-aminopurine and tryptophan fluorescence as probes in kinetic analyses of DNA polymerase. Biochemistry 41: 11226–11235. 12220188 10.1021/bi025837g

[27] Henzler-Wildman KA, Kern D (2007) Dynamic personalities of proteins. Nature 450: 964–972. 18075575 10.1038/nature06522

[28] Henzler-Wildman KA, Lei M, Thai V, Kerns SJ, Karplus M, et al (2007) A hierarchy of timescales in protein dynamics is linked to enzyme catalysis. Nature 450: 913–916. 18026087 10.1038/nature06407

[29] Boehr DD, Nussinov R, Wright PE (2009) The role of dynamic conformational ensembles in biomolecular recognition. Nat Chem Biol 5: 789–796. 19841628 10.1038/nchembio.232 PMC2916928

[30] Ma B, Nussinov R (2010) Enzyme dynamics point to stepwise conformational selection in catalysis. Curr Opin Chem Biol 14: 652–659. 20822947 10.1016/j.cbpa.2010.08.012 PMC6407632

[31] Cameron CE, Moustafa IM, Arnold JJ (2009) Dynamics: the missing link between structure and function of the viral RNA-dependent RNA polymerase. Curr Opin Struct Biol 19: 768–774. 19910183 10.1016/j.sbi.2009.10.012 PMC2787719

[32] Steitz TA (1993) DNA- and RNA-dependent DNA polymerases. Curr Opin Struct Biol 3: 31–38.

[33] Steitz TA, Steitz JA (1993) A general two-metal-ion mechanism for catalytic RNA. Proc Natl Acad Sci USA 90: 6498–6502. 8341661 10.1073/pnas.90.14.6498 PMC46959

[34] Castro C, Smidansky E, Maksimchuk KR, Arnold JJ, Korneeva VS, et al (2007) Two proton transfers in the transition state for nucleotidyl transfer catalyzed by RNA and DNA-dependent RNA and DNA polymerases. Proc Natl Acad Sci USA 104: 4267–4272. 17360513 10.1073/pnas.0608952104 PMC1838591

[35] Castro C, Smidansky ED, Arnold JJ, Maksimchuk KR, Moustafa I, et al (2009) Nucleic acid polymerses use a general acid for nucleotidyl transfer. Nature Structural and Molecular Biology 16: 212–218. 10.1038/nsmb.1540 PMC2728625 19151724

[36] McCammon JA, Gelin BR, Karplus M (1977) Dynamics of folded proteins. Nature 267: 585–590. 301613 10.1038/267585a0

[37] Karplus M, McCammon JA (2002) Molecular dynamics simulations of biomolecules. Nature Structural Biology 9: 646–652. 12198485 10.1038/nsb0902-646

[38] Mackerell AD (2004) Empirical force fields for biological macromolecules: overview and issues. J Comput Chem 25: 1584–1604. 15264253 10.1002/jcc.20082

[39] Mackerell AD (2005) Empirical Force Fields for Proteins: Current Status and Future Directions. Annual Reports in Computational Chemistry 1: 91–102.

[40] DeLano WL (2002) The PyMOL molecular graphics system. San Carlos (California): DeLano Scientific. Available: http://www.pymol.org. Accessed 26 February 2007.

[41] Case DA, Darden TA, Cheatham III TE, Simmerling CL, Wang J, et al.. (2008) AMBER 10. San Francisco: University of California.

[42] Jorgensen WL, Chandrasekhar J, Madura JD, Impey RW, Klein ML (1983) Comparison of simple potential functions for simulating liquid water. J Chem Phys 79: 926–935.

[43] Berendsen HJ, Postma JPM, Van Gunsteren WF, Dinola A, Haak JR (1984) Molecular-Dynamics with coupling to an external bath. J Chem Phys 81: 3684–3690.

[44] Ryckaert JP, Ciccotti G, Berendsen HJC (1977) Numerical integration of the cartesian equations of motion of a system with constraints: molecular dynamics of n-alkanes. J Comput Phys 23: 327–341.

[45] Darden T, York D, Pedersen L (1993) Particle mesh Ewald: an N·log(N) method for Ewald sums in large systems. J Chem Phys 98: 10089–10092.

[46] Shao J, Tanner SW, Thompson N, Cheatham TE (2007) Clustering molecular dynamics trajectories: 1. Characterizing the performance of different clustering algorithms. J Chem Theory Comput 3: 2312–2334. 26636222 10.1021/ct700119m

[47] Frisch MJ, Trucks GW, Schlegel HB, Scuseria GE, Robb MA, et al.. (2009) Gaussian 09, Revision A.1. Wallingford (Connecticut): Gaussian Inc.

[48] Kortus MG, Kempf BJ, Haworth KG, Barton DJ, Peersen OB (2012) A Template RNA Entry Channel in the Fingers Domain of the Poliovirus Polymerase. J Mol Biol 417: 263–278. 22321798 10.1016/j.jmb.2012.01.049 PMC3325025

[49] Yang X, Smidansky ED, Maksimchuk KR, Lum D, Welch JL, et al (2012) Motif D of Viral RNA-Dependent RNA polymerases Determines Efficiency and Fidelity of Nucleotide Addition. Structure 20: 1519–1527. 22819218 10.1016/j.str.2012.06.012 PMC3438331

[50] Mowat CG, Ruth Moysey R, Miles CS, Leys D, Doherty MK, et al (2001) Kinetic and Crystallographic Analysis of the Key Active Site Acid/Base Arginine in a Soluble Fumarate Reductase. Biochemistry 40: 12292–12298. 11591148 10.1021/bi011360h

[51] Schlippe YVG, Hedstrom L (2005) A twisted base? The role of arginine in enzyme-catalyzed proton abstractions. Archives of Biochemistry and Biophysics 433: 266–278. 15581582 10.1016/j.abb.2004.09.018

[52] Michielssens S, Moors SLC, Froeyen M, Herdewijn P, Ceulemans A (2011) tructural basis for the role of LYS220 as proton donor for nucleotidyl transfer in HIV-1 reverse transcriptase. Biophys Chem 157: 1–6. 21543151 10.1016/j.bpc.2011.03.009

