# Supplementary figures and images for: What Is the Role of Motif D in the Nucleotide Incorporation Catalyzed by the RNA-dependent RNA Polymerase from Poliovirus?
Source: PLoS Comput Biol. 2012 Dec 27;8(12):e1002851. doi: 10.1371/journal.pcbi.1002851 (PMC3531290; doi:10.1371/journal.pcbi.1002851)

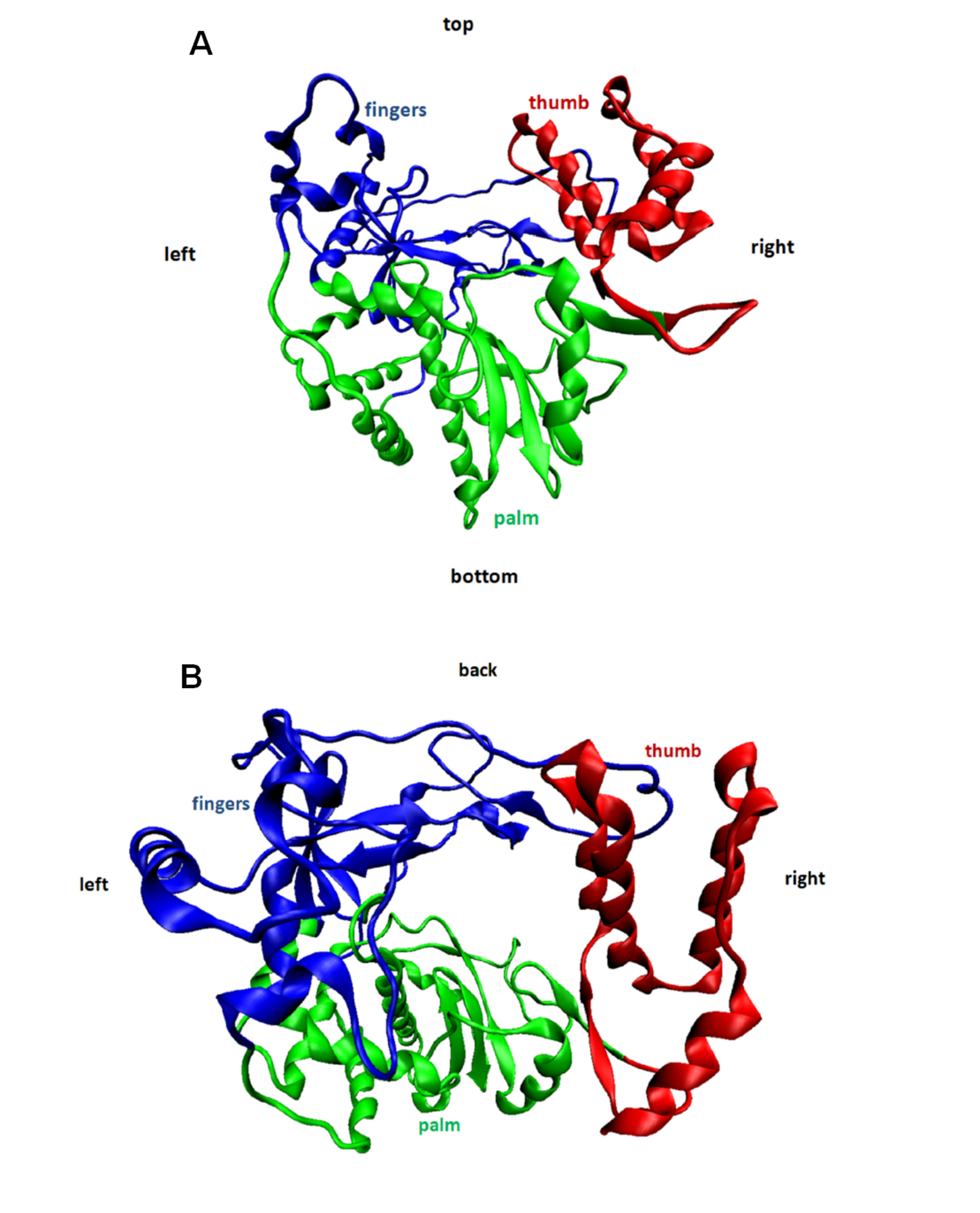

Supplement: Figure S1 — A front view (A) and a top view (B) of the cartoon representation of ligand-free PV 3Dpol (PDB code: 1RA6). Three domains of PV 3Dpol, fingers, palm and thumb, are depicted in blue, green and red, respectively. (TIFF) [file pcbi.1002851.s001.tiff]

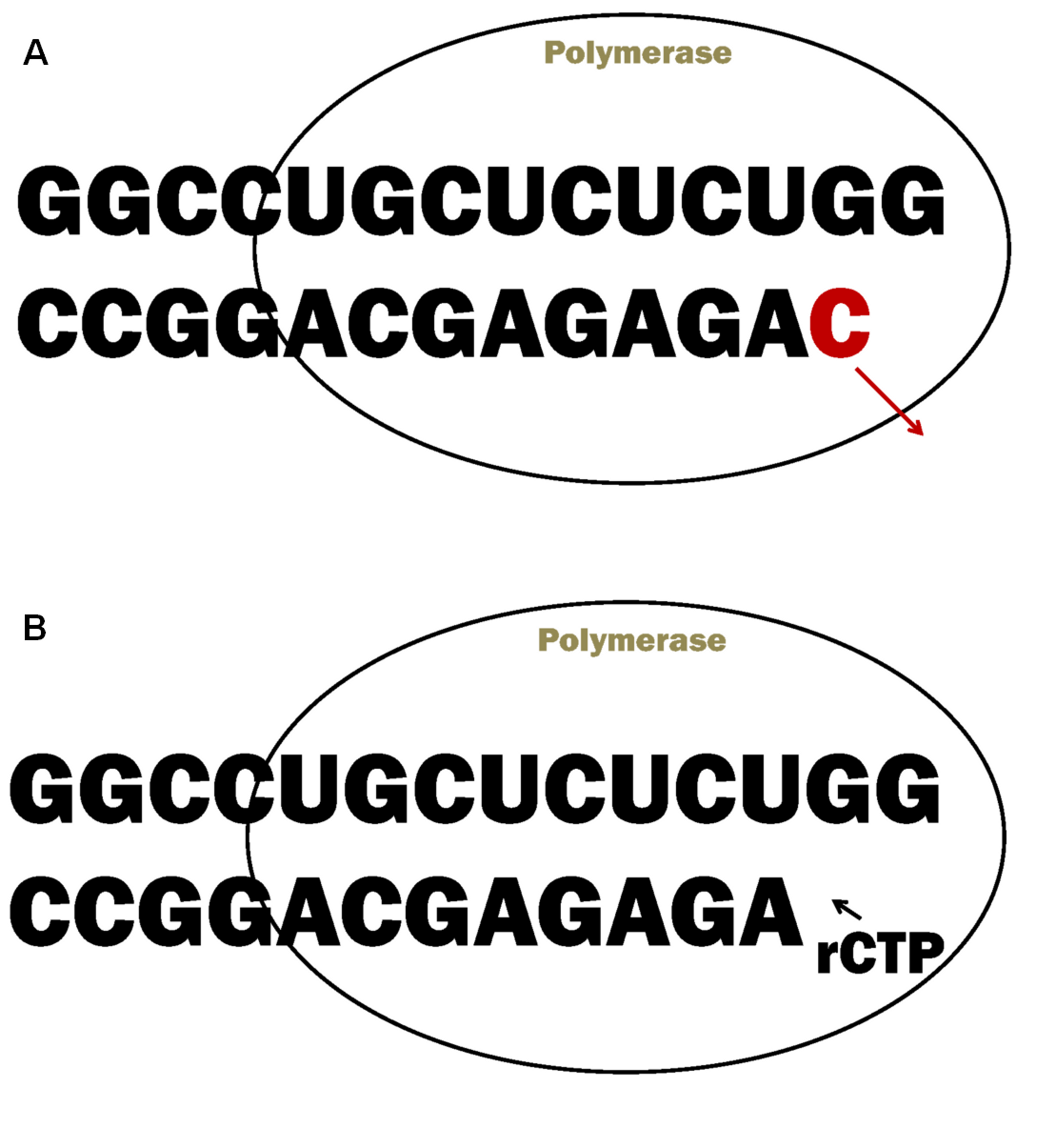

Supplement: Figure S2 — Nucleotide sequences of the RNA primer and template. Nucleotide sequences (A) of the RNA primer and template (PDB code: 3OL7) with the cytosine nucleotide (in red) that is to be deleted. The crystal structure (PDB code: 3OL7) reflects a state of the post-chemistry step of the nucleotide incorporation. After deleting the cytosine nucleotide from the RNA sequence in (A), the final nucleotide sequences (B) of the RNA primer and template were retained in the structure into which rCTP molecule was built. (TIFF) [file pcbi.1002851.s002.tiff]

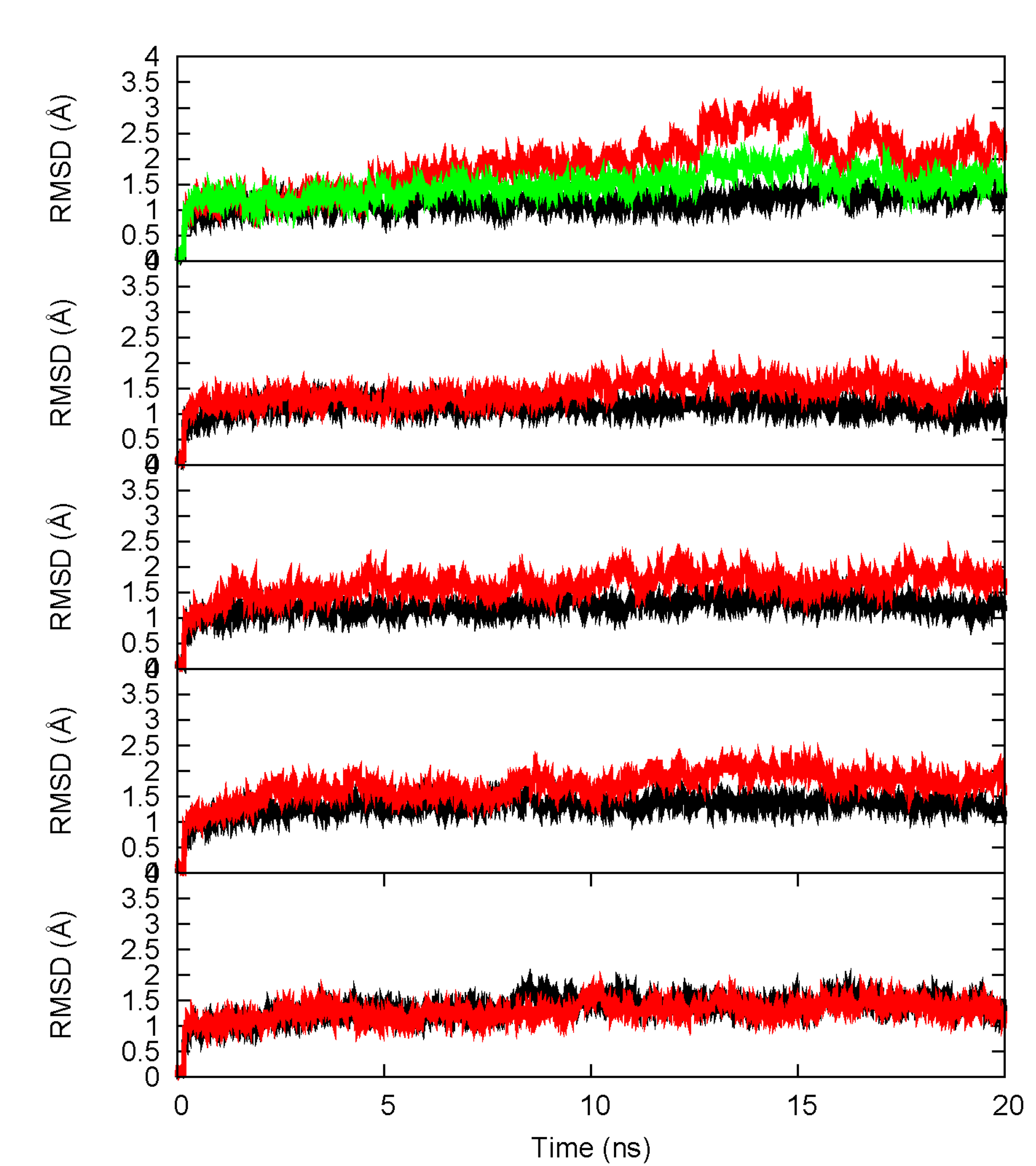

Supplement: Figure S3 — Backbone heavy-atom RMSD curves of PV 3Dpol. The results were calculated from five independent MD simulations of the PV 3Dpol apo (red) and complex (black) structures. On the top-box, the backbone heavy- atom RMSD values were calculated from the first MD simulation for the apo form of PV 3Dpol excluding two segments (residues 210–220 and 380–461) and were plotted against simulation time (green curve). (TIFF) [file pcbi.1002851.s003.tiff]

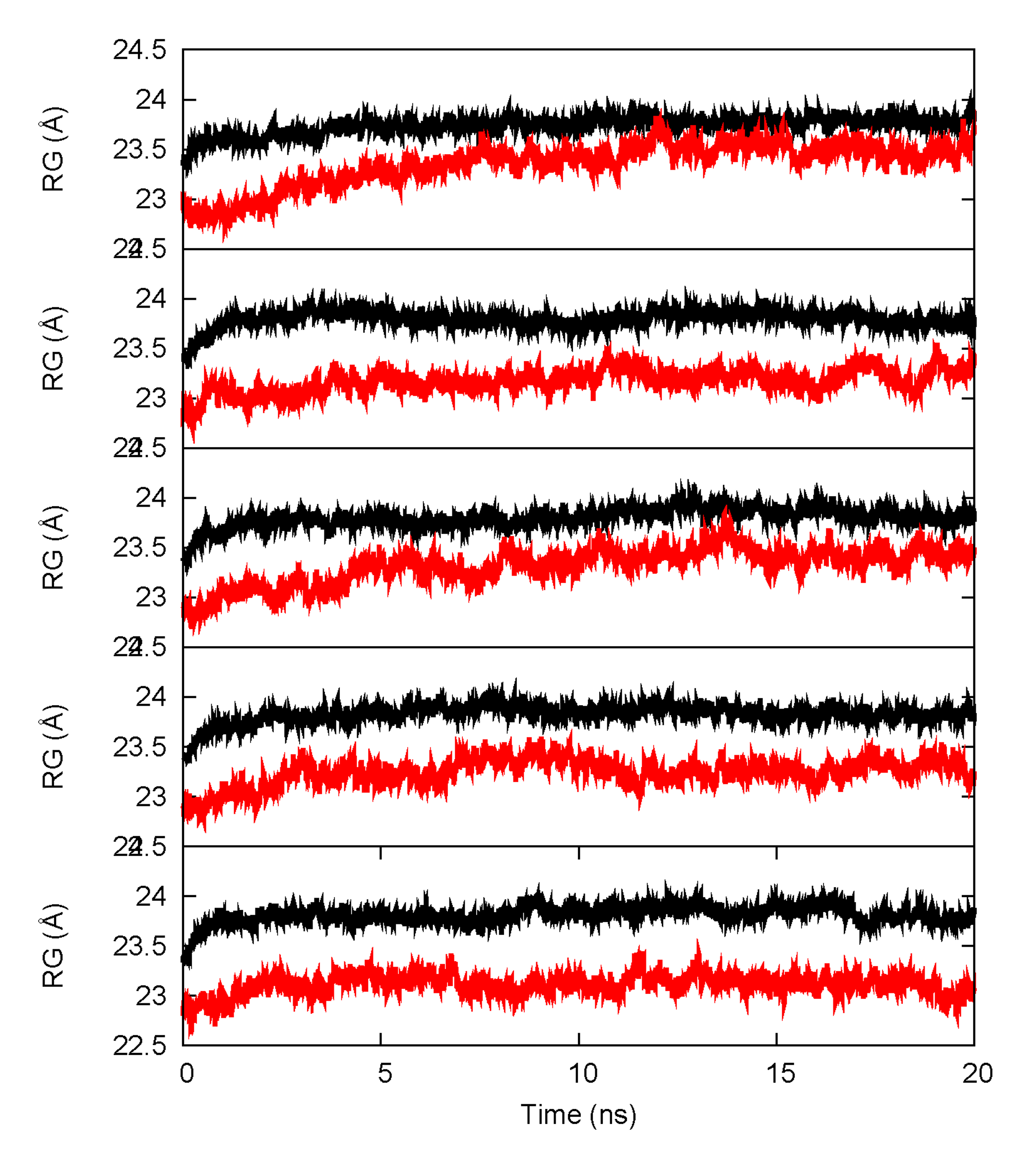

Supplement: Figure S4 — Radius of gyration (RG) curves for the backbone heavy atoms of PV 3Dpol in the apo (red) and complex (black) forms. The results were calculated from five independent MD simulations of the PV 3Dpol apo and complex structures. (TIFF) [file pcbi.1002851.s004.tiff]

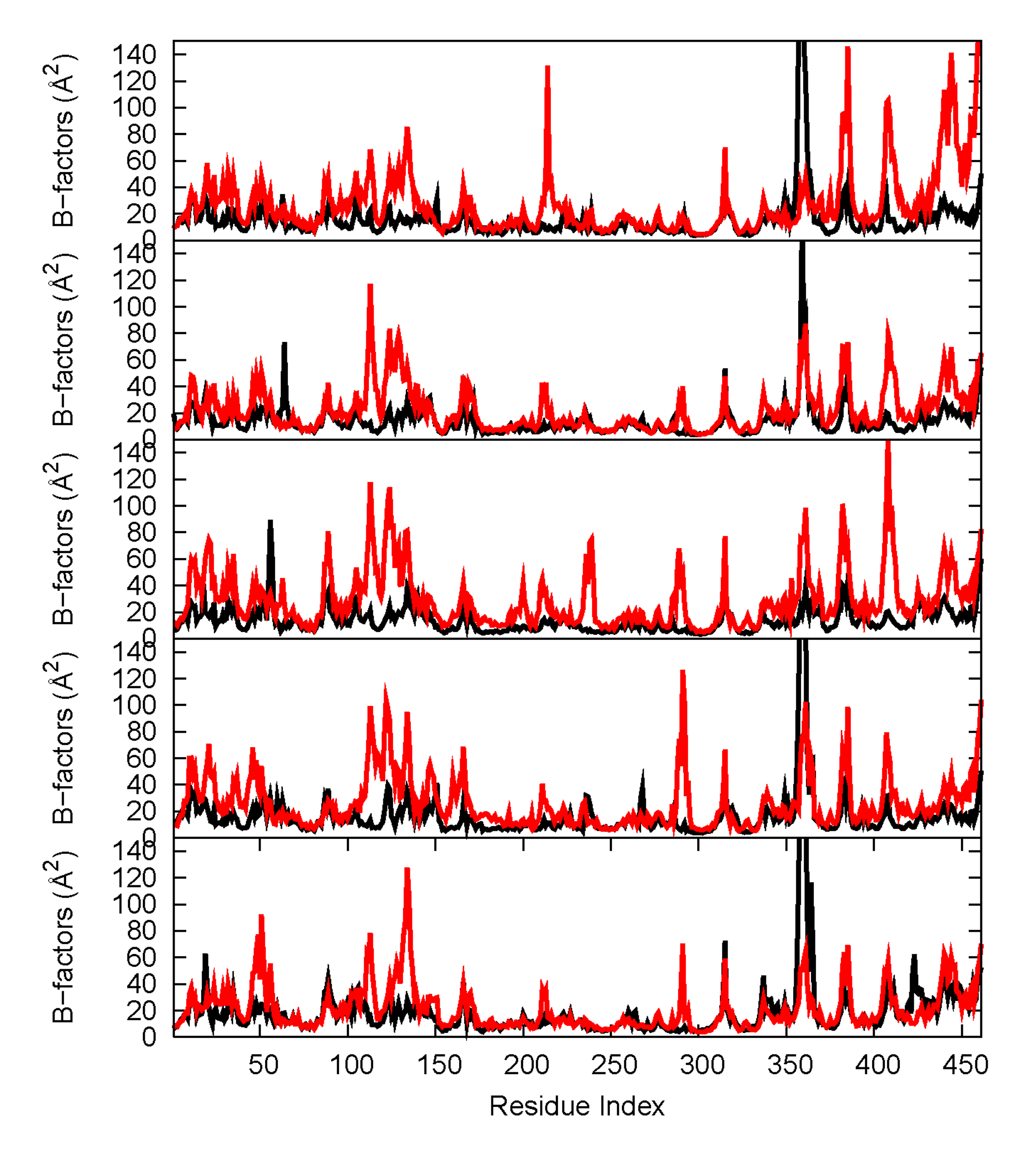

Supplement: Figure S5 — B-factors of the backbone alpha carbons for PV 3Dpol in the apo (red) and complex (black) forms. The results were obtained from five independent MD simulations of the PV 3Dpol apo and complex structures respectively. (TIFF) [file pcbi.1002851.s005.tiff]

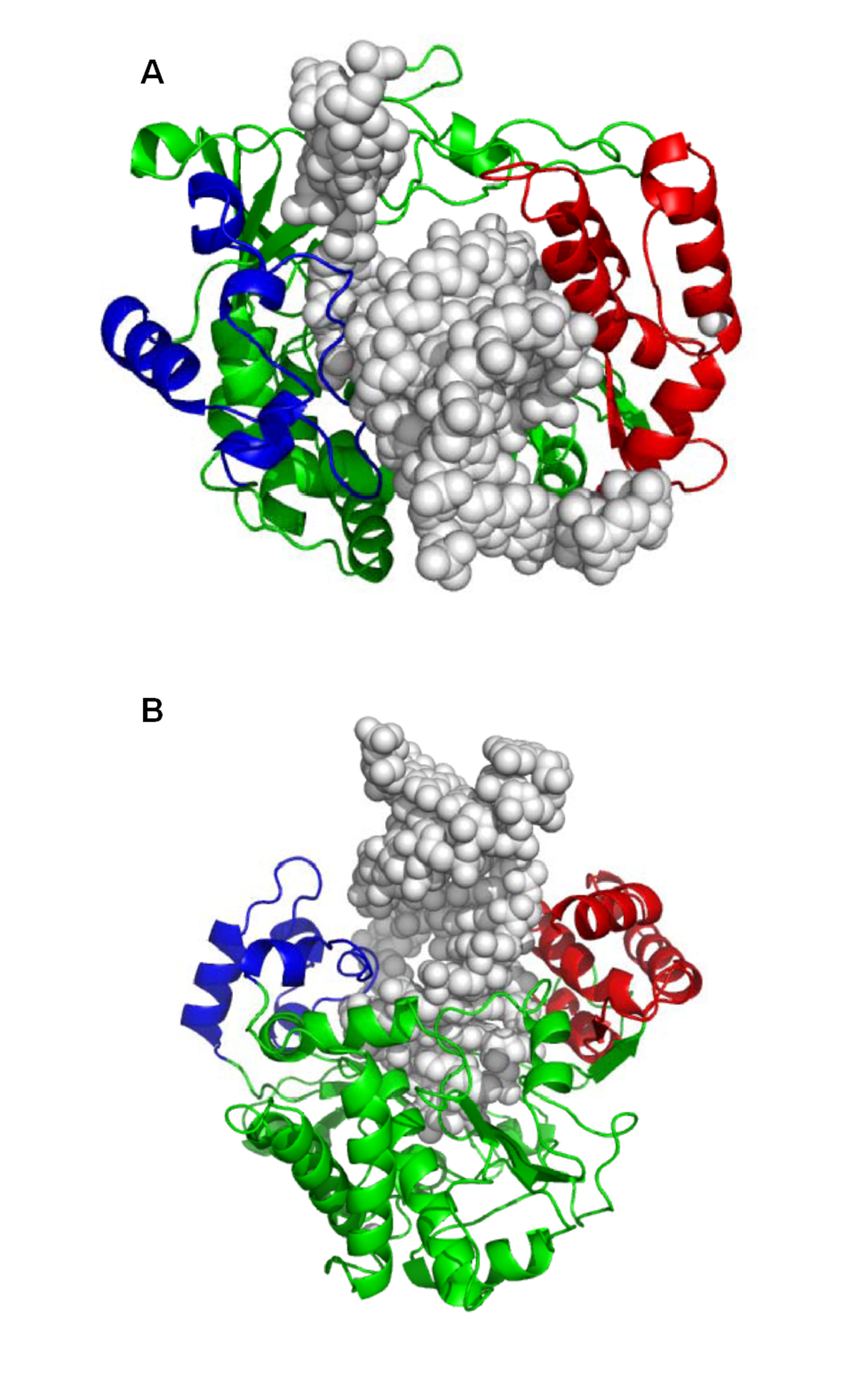

Supplement: Figure S6 — A top view (A) and a side view (B) of the 3Dpol-RNA-rCTP complex structure with the cartoon representation of protein (green) and VDW representation of RNA (white). Segments from the pinky finger and the thumb domain are indicated in blue and red respectively. (TIFF) [file pcbi.1002851.s006.tiff]

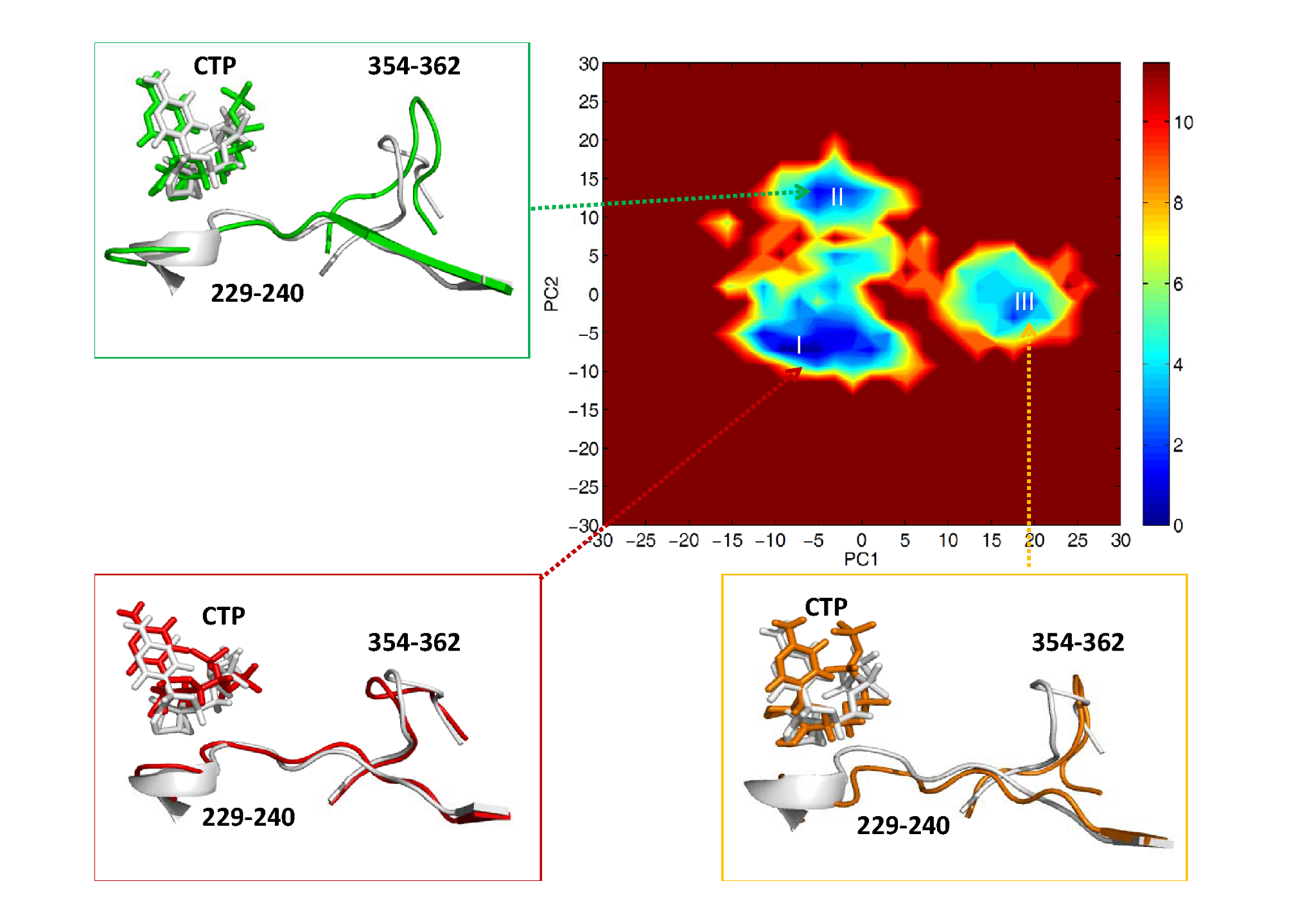

Supplement: Figure S7 — Free energy (in unit of kcal/mol) profile of (PC1, PC2) for PV 3Dpol in the complex form. In the three states (I, II, III), the stick representation of CTP, and the cartoon representation of motif A and residues 354–362 of motif D, are depicted in red, green and orange respectively, and the crystal structure is indicated in white gray. (TIFF) [file pcbi.1002851.s007.tiff]

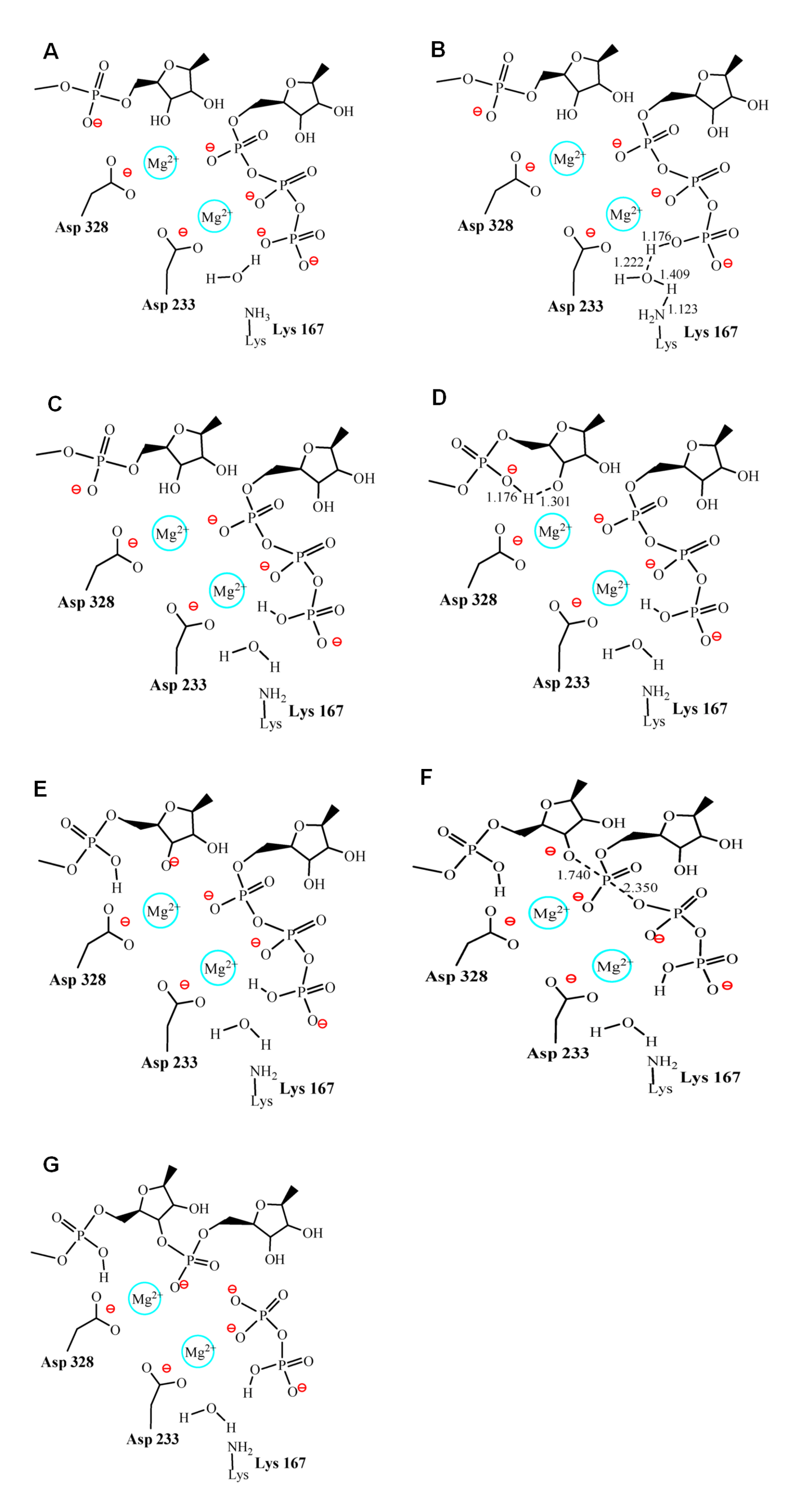

Supplement: Figure S8 — Illustration of the reactant (A), the transition state (B), the intermediate state (C), the transition state (D), the intermediate state (E), the transition state (F), and the product (G) in the nucleotidyl transfer. The results were obtained from the quantum mechanics calculations carried out on the truncated system with Lys167. The subscript of the intermediate state (IM) indicates the number of a state, and the subscript of the transition state (TS) represents the transition from one state to another. (TIFF) [file pcbi.1002851.s008.tiff]

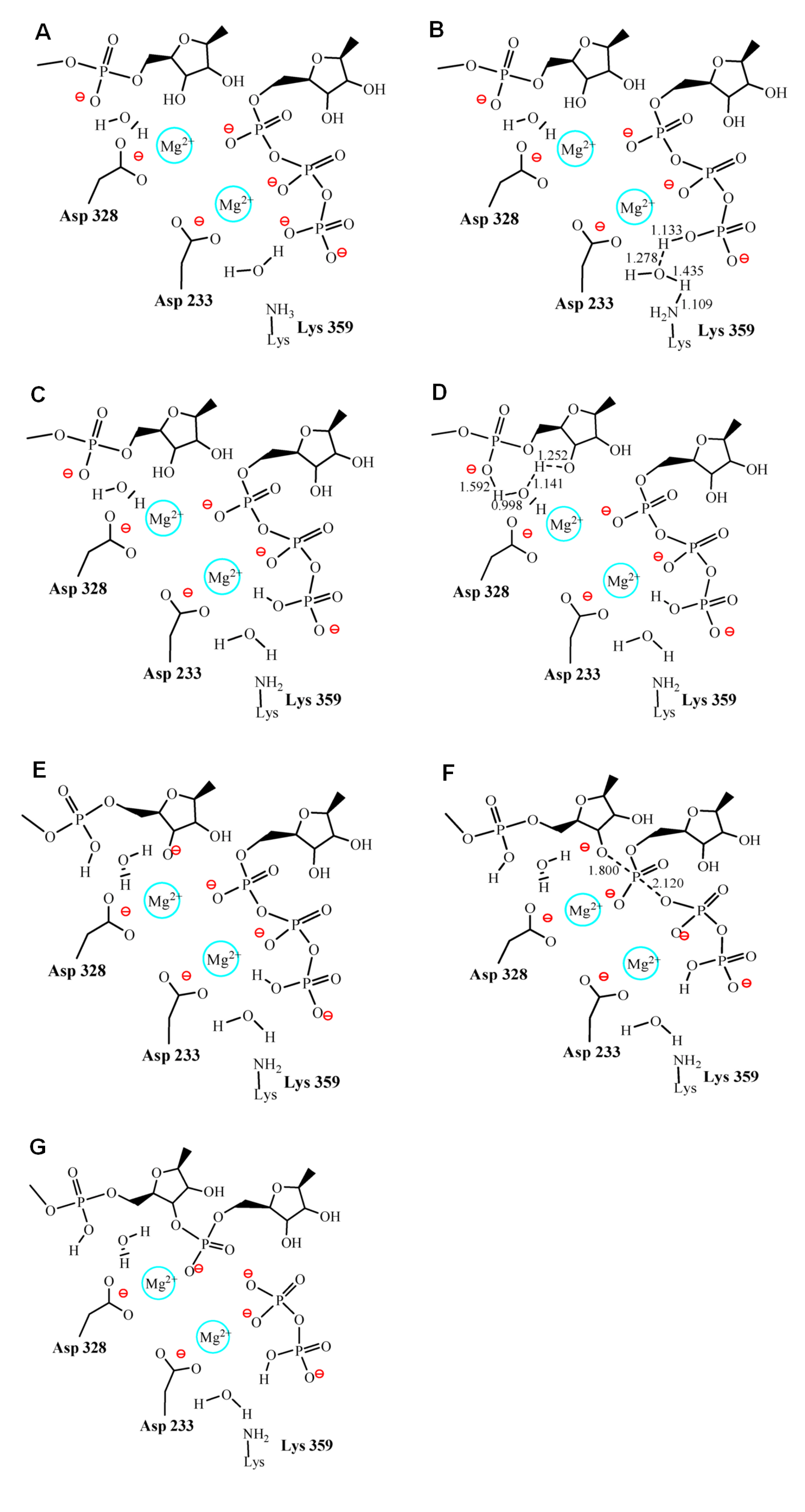

Supplement: Figure S9 — Illustration of the reactant (A), the transition state (B), the intermediate state (C), the transition state (D), the intermediate state (E), the transition state (F), and the product (G) in the nucleotidyl transfer. The results were obtained from the quantum mechanics calculations carried out on the truncated system with Lys359. The subscript of the intermediate state (IM) indicates the number of a state, and the subscript of the transition state (TS) represents the transition from one state to another. (TIFF) [file pcbi.1002851.s009.tiff]

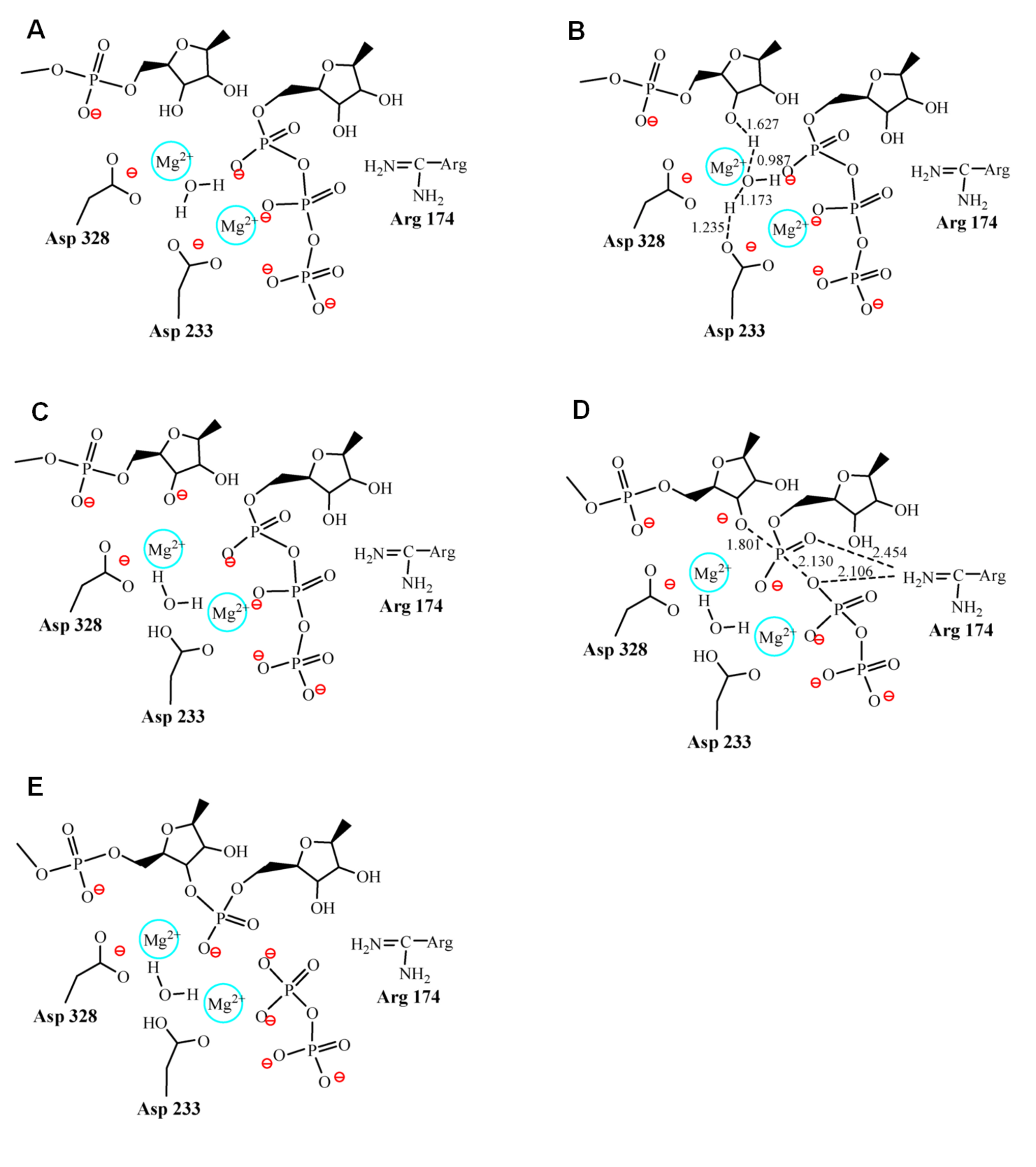

Supplement: Figure S10 — Illustration of the reactant (A), the transition state (B), the intermediate state (C), the transition state (D), and the product (E) in the nucleotidyl transfer. The results were obtained from the quantum mechanics calculations carried out on the truncated system with Arg174. The subscript of the intermediate state (IM) indicates the number of a state, and the subscript of the transition state (TS) represents the transition from one state to another. (TIFF) [file pcbi.1002851.s010.tiff]
